# Fluoride Varnish for Caries Prevention in Preschoolers: An Overview of Reviews

**DOI:** 10.1111/cdoe.70032

**Published:** 2025-11-20

**Authors:** Flávia Macedo Couto, Fernanda Santos de Oliveira Sousa, Izabel Monteiro Dhyppolito, Fernanda Barja‐Fidalgo, Ana Paula Pires dos Santos, Paulo Nadanovsky

**Affiliations:** ^1^ Department of Community and Preventive Dentistry, Faculty of Dentistry Rio de Janeiro State University Rio de Janeiro Brazil; ^2^ Department of Epidemiology, Institute of Social Medicine Rio de Janeiro State University Rio de Janeiro Brazil; ^3^ Department of Pediatric Dentistry and Orthodontics, School of Dentistry Universidade Federal do Rio de Janeiro Rio de Janeiro Brazil; ^4^ Department of Epidemiology and Quantitative Methods in Health, National School of Public Health Oswaldo Cruz Foundation Rio de Janeiro Brazil

**Keywords:** child preschool, dental caries, fluorides, systematic review, topical

## Abstract

**Objectives:**

This study aimed to overview the available evidence from systematic reviews (SRs) on the effects of fluoride varnish (FV) for caries prevention in preschoolers.

**Methods:**

Systematic reviews, with or without meta‐analyses, of randomised controlled trials (RCTs) and quasi‐randomised trials evaluating the use of FV in preschoolers to prevent dentin caries compared to placebo, standard care, or no intervention were included. The search was last updated in July 2025 across eight electronic databases. Two researchers independently assessed eligibility and extracted data, resolving disagreements by discussion or with a third researcher if needed. The methodological quality, risk of bias, and certainty of evidence of the SRs were assessed using AMSTAR‐2, ROBIS, and GRADE, respectively. The results were synthesised descriptively.

**Results:**

Fourteen SRs published between 2001 and 2023 were included. Six SRs reported insufficient evidence to conclude on the effectiveness of FV; six concluded that FV is effective; and two suggested that FV provides a probably irrelevant clinical benefit. One SR had a high methodological quality, two had low, and 11 were rated as critically low. The risk of bias was considered low in three SRs and high in 11. The certainty of evidence ranged from moderate to very low, with the risk of bias being the criterion that most contributed to downgrading it.

**Conclusions:**

Most systematic reviews on the effectiveness of fluoride varnish in preventing caries in preschoolers are of critically low quality and high risk of bias, with conflicting findings. Systematic reviews that included more recent studies with lower risk of bias indicated that fluoride varnish provides relatively limited or no additional benefit to children who use fluoride toothpaste. Therefore, the routine application of fluoride varnish in preschoolers should be reconsidered.

## 1 Introduction

Systematic reviews (SRs) gather evidence from selected primary studies to address specific research questions [1]. The number of published SRs has been increasing [2], partly due to their higher citation rates, which may enhance a journal's impact factor [3]. In dentistry, while many studies have been published, their methodological quality (MQ) raises concerns about whether they actually provide reliable evidence [4]. Some SRs may be outdated or of inadequate quality [5]. The high number of SRs on a given topic, often with conflicting conclusions, can confuse readers and clutter the scientific ecosystem. This may lead to clinical practices based on personal preference rather than high‐quality, up‐to‐date evidence, which should form the basis of clinical recommendations [6].

Various clinical practice recommendations suggest FV applications for caries prevention in preschoolers [7]. These recommendations differ in frequency and the population targeted, depending on caries risk [7]. While such variations are appropriate, they should be grounded in the same best available evidence [7]. However, more recent trials on FV [8, 9, 10, 11, 12] have not found a clear anticaries benefit, even in high caries risk populations [9, 11, 12]. Several SRs [13, 14, 15, 16, 17, 18, 19, 20, 21] have evaluated the effectiveness of FV, with more recent SRs offering conflicting conclusions: some support its effectiveness in reducing caries [20, 21] while others find no clinically relevant benefit [13, 14]. These conflicting conclusions may create uncertainty for clinicians, underscoring the need for a synthesis and critical appraisal of existing SRs, and highlighting the importance of conducting an overview of the topic.

When relying on a SR as the scientific basis for health practices, various factors must be considered, including methodological quality, risk of bias (in both the SR and its included studies), publication date, and the certainty of evidence. Although several systematic reviews have investigated the anticaries benefit of fluoride varnish in preschool children, no overview to date has critically appraised these reviews as a collective body of evidence. Therefore, this study aimed to overview the available evidence from SRs on the anticaries effects of FV applications in preschoolers.

## 2 Material and Methods

This overview of reviews was planned and conducted according to the Cochrane Handbook for Systematic Reviews of Interventions [1, 22]. The report followed the Preferred Reporting Items for Overviews of Reviews (PRIOR) Statement [23]. The protocol of this study was prospectively registered in Open Science Framework (https://osf.io/kc2xn/) and is publicly available.

### 2.1 Eligibility Criteria

Systematic reviews with or without meta‐analyses (MA) of randomised and quasi‐randomised controlled trials (RCT) that assessed the use of FV for caries prevention in preschoolers (i.e., children up to 71 months of age) regardless of fluoride concentration, fluoride agent, application frequency, or commercial brand were included. A study was considered a SR if it aimed to summarise research through a systematic search and selection process of primary research, adhering to predetermined eligibility criteria.

The comparisons considered were placebo, standard care, or no intervention. In the case of multiple versions of a SR, the most recent publication was included. Systematic reviews that evaluated different age groups were included if the data were available separately for preschoolers.

The primary outcome was caries lesions at the dentin level in the primary dentition assessed by any caries index at the surface, tooth, or individual levels. The secondary outcomes were any adverse effects reported by dentists, children, or guardians, and costs.

### 2.2 Information Sources

The electronic search was conducted in March 2023 and updated in July 2025 with no restrictions on language or publication date, across the following databases: MEDLINE via PubMed, Web of Science, Cochrane Database of Systematic Reviews (CDSR), Scopus, EMBASE, BBO and LILACS via BVS, Epistemonikos, and Health Evidence.

Systematic review registries were searched through the International Prospective Register of Systematic Reviews (PROSPERO) to identify any unpublished or undetected SRs in the database search.

Additionally, the references of the included SRs were checked for potentially eligible studies.

### 2.3 Search Strategy

The search strategy was initially developed for PubMed and then adapted for use in other databases (Appendix S1).

### 2.4 Selection Process

EndNote Web (Clarivate Analytics, Philadelphia, Pennsylvania, USA) was used to store references and remove duplicates. The references were then exported from EndNote to the Rayyan web and mobile application for SRs (https://www.rayyan.ai) [24]. Two researchers independently evaluated the eligibility of titles and abstracts. If the title and abstract lacked sufficient information to determine inclusion, the full article was read. Disagreements between the two researchers were resolved by discussion. A third researcher aided the decision when disagreements remained.

### 2.5 Reviewers' Training

Before data collection, reviewers underwent training on data extraction and application of the tools. They then independently collected data and evaluated three SRs of dental interventions that were not eligible for inclusion in this overview. The reviewers discussed the collected data and reached a consensus on the application of the tools.

### 2.6 Data Extraction, Methodological Quality Assessment, and Risk of Bias Assessment

Two reviewers independently collected and registered the characteristics of the SRs in a standardised spreadsheet (Appendix S2). Data were collected only on FV and the eligible participants and comparisons. Disagreements were resolved by discussion between the two reviewers, with a third researcher consulted if disagreements persisted. In cases where information was missing or unclear, the primary studies were reviewed. If they remained unsolved, the review authors were contacted for clarification.

The reviewers evaluated the methodological quality (MQ) and risk of bias (RB) using the AMSTAR‐2 [25] and ROBIS [26] tools, respectively. Disagreements were resolved through discussion between the two reviewers or with the mediation of a third reviewer. In case of doubt or when information was unavailable, the reviewers attempted to contact the review authors. Overall confidence in the findings of the SR, according to AMSTAR‐2, was rated based on the identification of critical domains adapted from the tool's suggested critical items. Prior to the assessment, the authors established the following items as critical: adequacy of the literature search, justification for excluding individual studies, risk of bias from individual studies included in the review, appropriateness of meta‐analytical methods, consideration of RB when interpreting the findings of the review, and assessment of the presence and likely impact of publication bias.

### 2.7 Data Synthesis

Data on the characteristics of the SRs included were summarised narratively and in tables.

### 2.8 Certainty of Evidence

The certainty of evidence is presented according to the GRADE approach [27, 28]. When available in the included SR, GRADE assessments were extracted and reported. In the absence of such assessments, GRADE evaluations based on the systematic reviews' report were conducted by the authors. For SR with Network meta‐analysis (NMA), the CINeMA assessment [29] was collected.

## 3 Results

**FIGURE 1:**
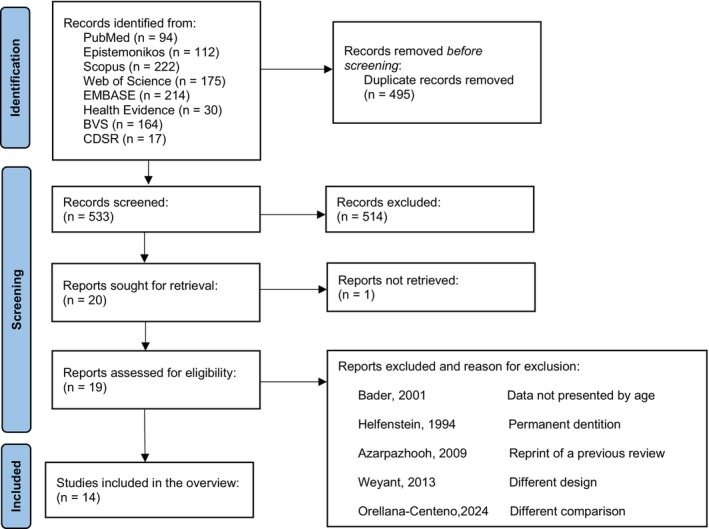
Identification of studies via databases and registers.

A total of 1028 references were identified, and after removing the duplicates, 533 records were screened. Out of the 20 considered relevant for full‐text reading, one full‐text could not be retrieved. After the exclusion of five studies, 14 reviews were included [13, 14, 15, 16, 17, 18, 19, 20, 21, 30, 31, 32, 33, 34]. Seventy‐two protocols were identified through PROSPERO, screened, and no additional study was identified (Figure 1).

Five [13, 14, 15, 20, 21] of the 14 reviews performed MA, including two NMAs [20, 21]. The SRs was published from 2001 to 2023 and included from one to 22 studies (Table 1).

**TABLE 1:** Characteristics of included systematic reviews on the anticaries effect of fluoride varnish for preschoolers.

Author, year	Title	Number of included studies/participants	Search date	Databases included	Study design	Participants	Comparisons
Bader et al., 2001 [17]	A Systematic Review of Selected Caries Prevention and Management Methods	1 study/NI	From 1966 to October of 1999	MEDLINE, EMBASE and CENTRAL	SR	Primary teeth	No intervention
Rozier, 2001 [31]	Effectiveness of Methods Used by Dental Professionals for the Primary Prevention of Dental Caries	7 studies/NI	From 1966 to 2000	MEDLINE	SR	Primary teeth	No intervention
Petersson et al., 2004 [32]	Professional Fluoride Varnish Treatment for Caries Control: A Systematic Review of Clinical Trials	3 studies/4732 participants	From 1966 to November 2001. Updated in April 2003	MEDLINE and Cochrane library	SR	Primary teeth	Placebo, no interventionor other fluoride
Azarpazhooh and Main, 2008 [18]	Fluoride Varnish in the Prevention of Dental Caries in Children And Adolescents: A Systematic Review	3 studies/1951 participants	From 2000 to 2007	Ovid MEDLINE, CINAHL, CENTRAL, CDSR, DARE, EMBASE, Health and Psychosocial Instruments, HealthSTAR, International Pharmaceutical Abstracts, Journals@Ovid and ACP Journal Club	SR	0–18 years	No intervention
Carvalho et al., 2010 [19]	Fluoride Varnishes and Caries Incidence Decrease in Preschool Children: A Systematic Review	8 studies/2501 participants	Up to December 2008	BBO and LILACS, Medline and the Cochrane Library	SR	Children up to 6 years of age	No intervention
Marinho et al., 2013 [15]	Fluoride Varnishes for Preventing Dental Caries in Children and Adolescents	22 studies/12 455 participants randomised (9595 used in analyses)	Up to 13 May 2013	The Cochrane Oral Health Group's Trials Register; CENTRAL (The Cochrane Library); MEDLINE via OVID; EMBASE via OVID; CINAHL via EBSCO; LILACs via BIREME Virtual Health Library; BBO via BIREME Virtual Health Library; ProQuest Dissertations and Theses; Web of Science Conference Proceedings	SR MA	Children or adolescents aged 16 or less at the start of the study	Placebo or no intervention
Twetman and Dhar, 2015 [30]	Evidence of Effectiveness of Current Therapies to Prevent and Treat Early Childhood Caries	7 studies/3485 participants	Between 2007 and April 2014	PubMed and Cochrane library	SR	Children before 3 years of age	Placebo, oral health promotion or no intervention
Mishra et al. 2017 [16]	Role of Fluoride Varnish in Preventing Early Childhood Caries: A Systematic Review	17 studies/13 583 participants	Up to 2015	PubMed/Medline, Cochrane, EMBASE and IRIS database WHO	SR	Children/primary teeth	Placebo, oral health promotion or no intervention
Sousa et al., 2019 [14]	Fluoride Varnish and Dental Caries in Preschoolers: A Systematic Review and Meta‐Analysis	20 studies (19 in the qualitative analysis and 17 in at least one meta‐analysis)/16 877 children randomised, and 13 658 included in the analyses.	Up to 2018	CENTRAL, MEDLINE via PubMed, Web of Science, EMBASE, SCOPUS, LILACS, and BBO. Abstracts of the International Association for Dental Research (2001–2018) and the European Organisation for Caries Research (1998–2018), Open Grey, EThOS, the New York Academy of Medicine (GreyLit Report), and Banco de Teses CAPES. Current Controlled Trials, ClinicalTrials.gov, EU Clinical Trials Register, Australia New Zealand Clinical Trials Registry, and Registro Brasileiro de Ensaios Clínicos.	SR MA	Children up to 71 months of age (preschoolers)	Placebo, usual care, or no intervention
Yu et al.,2021 [13]	The Additional Benefit of Professional Fluoride Application for Children as an Adjunct to Regular Fluoride Toothpaste: A Systematic Review and Meta‐Analysis	5 studies/4370 participants	Up to February 2020	PubMed, CENTRAL, Embase and Google Scholar. Reference lists of eligible trials, relevant systematic and narrative reviews. Hand searching was performed for ten relevant dental journals	SR MA	Children aged 16 or younger at baseline (including children with primary, mixed or permanent dentition)	Self‐applied regular fluoride toothpaste alone, with a fluoride concentration of 1000 ppm or above
Manchanda et al., 2021 [20]	Topical Fluoride to Prevent Early Childhood Caries: Systematic Review With Network Meta‐Analysis	13 studies/10 279 participants	Up to July 2020	Medline (via Ovid), PubMed, Embase (via Ovid), Scopus, Lilacs, CINAHL, Web of Science, and Cochrane Library. The reference list of the included studies and previous published systematic reviews were searched manually. Additional www.clinicaltrials.gov and http://opengrey.eu were searched.	SR NMA	Children younger than 6 years of age	Any other form/concentration of topical fluoride or placebo or no intervention
Muantenu et al., 2022 [33]	Review of Professionally Applied Fluorides for Preventing Dental Caries in Children and Adolescents	3 studies/846 participants	Papers published between 2000 and 2021	Pubmed, Google Scholar, Cochrane Library, and ResearchGate	SR	Children and adolescents, treated in a dental care setting	No professional treatment or other preventive treatments
He et al., 2023 [21]	Clinical Interventions With Various Agents to Prevent Early Childhood Caries: A Systematic Review With Network Meta‐Analysis	34 studies (34 in the qualitative analysis and 33 in network meta‐analysis (NMA))/Included in FV intervention group in NMA: 2584 children (caries increment) and 1280 children (caries incidence)	Up to June 2021	Medline (via PubMed), Embase (via Ovid), and CENTRAL	SR	Children up to 72 months old, with at least one caries‐free tooth (dmft = 0) and without serious systemic diseases	Placebo control, active control or no intervention
Rup et al., 2023 [34]	Caries Incidence After Professional Fluoride Treatment: A Systematic Review	3 study/NI	Up to February 2022	Medline via PubMed, Embase, LILACS, CENTRAL, ClinicalTrials.gov	SR	Adolescents and children without restriction of age, gender, or ethnicity	No intervention or use of fluoride toothpaste

Abbreviations: MA, meta‐analysis; NI, no information; NMA, network‐ meta‐analysis; SR, systematic review.

The SRs included a total of 55 primary studies, with 26 overlapping at least once and 29 appearing in only one SR. One of the most cited studies was Holm, 1979 [35] which was included in eight SR [14, 15, 16, 19, 20, 21, 31, 32]. The results from Lawrence et al. [36] also appeared in eight SR [14, 15, 16, 18, 20, 21, 30, 34], with one [18] SR collecting the data from a conference prior to the publication. This was followed by Weintraub et al. [37], included in seven SR [14, 15, 16, 18, 19, 20, 21]. Grodzka et al., 1982 [38] was included in six SR [14, 16, 19, 21, 31, 32] as well as [13, 14, 20, 21, 30, 33] Oliveira et al., 2014 [10] (Appendix S3).

Among the SRs [13, 14, 15, 16, 19, 33] that reported the product formulation, 5% sodium fluoride was the most frequently included, with 0.9% difluorosilane also mentioned. The most commonly used commercial brand in RCTs was Duraphat.

Some earlier SRs [16, 17, 19, 30, 31, 32], published up to 2017, suggested that the evidence for the anticaries effect of FV was limited or inconclusive. Two SRs [13, 14], published in 2019 and 2021, indicated that FV had minimal impact on caries incidence, while six others [15, 18, 20, 21, 33, 34], published between 2008 and 2023, suggested that FV was effective in preventing caries (Table 2).

**TABLE 2:** Findings from the systematic reviews included in the overview.

Author, year	Outcome	Summary of findings for the review	Certainty of evidence^a^	Review conclusions
Bader et al., 2001 [17]	Incidence of Coronal Carious Lesions in Primary Teeth	Out of nine evaluations for the efficacy of fluorides for the prevention of carious lesions one study examined effects on primary teeth and showed significant effects The increment in experimental group was 1.7 and in control group it was 2.3 (dfs proximal surfaces only), with a significant 25% reduction and a NNT of 3.5.	⊕⊝⊝⊝ Very low	Insufficient evidence to determine the anticaries benefit of FV in preschoolers
Rozier, 2001 [31]	Effectiveness of fluoride varnish in inhibiting caries in primary teeth. The primary measure of outcome is the Prevented Fraction (PF), or the proportional reduction in dental caries between experimental and control participants	The SR included seven studies. The PF in the included studies varied from 5.3% to 43.8%. The NNT was expressed for three of the included studies and was 1.2, 1.5, and 4.3. Only two out of six were RCT with inconsistent findings. One found the higher preventive fraction (43.8%) and had statistically significant results, while the other RCT was one of the five included studies with no statistically significant	⊕⊝⊝⊝ Very low	Inconclusive evidence to determine the anticaries benefit of FV in preschoolers
Petersson et al., 2004 [32]	Coronal caries increment in the deciduous dentition	The SR included three studies that were graded as A to C according to predetermined criteria for methodology and performance. Two were graded as level C (low value as evidence) and had no statistical significance. Their results were a dmfs increment of 1.3 and 6.4 with a dmft increment of 1.4 and 6.7. One was graded as B (moderate value as evidence), had a statistically significant result in caries reduction with a PF of 44% and had a dmfs of 2.1 and dmft increment of 3.7	⊕⊝⊝⊝ Very low	Inconclusive evidence to determine the anticaries benefit of FV in preschoolers
Azarpazhooh and Main, 2008 [18]	Efficacy of fluoride varnish	The SR included seven articles in total with three regarding the preventive effect in preschoolers. The results of two primary studies were described in the SR text, one of those conducted in children from 6 months to 5 years of age. The study was pointed as second strongest study in this series of all seven with a level of evidence I and grade of recommendation A, meaning the study was assessed as a properly RCT representing good evidence to recommend for the clinical preventive action. In this primary study dmfs increment was 10.17 ± 0.46 for the FV group versus 13.47 ± 0.90 for the control group, *p* = 0.047, yielding a percentage reduction of 24% + 5.3% in First‐Nation and non‐First‐Nation children combined	⊕⊝⊝⊝ Very low	There is clear evidence of the anticaries benefit of FV in preschoolers
Carvalho et al., 2010 [19]	Incidence of caries, given the presence of a cavitated lesion (level of detection C2—enamel caries, or C3dentine caries) in primary dentition (dmfs)	Most of the eight included trials had problems in terms of design according to the Jadad's scale assessment. The number of cavitated carious surfaces increased varying from 0.5 to 6.3 in the test group and from 1.4 to 6.7 in the control group. The difference between groups in caries increment in cavitated lesions varied from 0.30 to 1.64. The PF varied from 5% to 63%	⊕⊝⊝⊝ Very low	Inconclusive evidence to determine the anticaries benefit of FV in preschoolers
Marinho et al., 2013 [15]	d(e/m)fs increment (PF)	Ten trials reported data which allowed the calculation of the d(e/m)fs PF. The pooled estimate of d(e/m)fs PF was 0.37 (95% CI 0.24 to 0.51; *p* < 0.0001), suggesting a substantial benefit of FV in the primary dentition. There was statistically significant heterogeneity between trials (*χ* ^2^ = 21.83 on 9 degrees of freedom, *p* = 0.009, *I* ^2^ = 59%). A 95% prediction interval for the pooled trials was calculated and ranged from‐0.01, 0.76, indicative of a benefit of FV in the most part	⊕⊕⊕⊝ Moderate	FVs applications is associated with a substantial reduction in caries increment
	d(e/m)ft. increment (PF)	Two trials reported data which allowed the calculation of the d(e/m)fs PF. The fixed‐effect pooled estimate was 0.65 (95% CI 0.48 to 0.82; *p* < 0.0001), suggesting a substantial benefit of FV in the primary dentition. There was no evidence of statistically significant heterogeneity between trials (*χ* ^2^ = 0.04 on 1 degree of freedom, *p* = 0.83, *I* ^2^ = 0%)	⊕⊕⊕⊝ Moderate	
	Developing one or more new caries (d(e/m)ft)	Five trials reported results on the proportion of children developing one or more new caries (whole tooth) in the permanent dentition; five in the primary dentition. There was no evidence of effectiveness of FV in the primary dentition (RR = 0.81, 95% CI 0.62 to 1.06, *p* = 0.13)	⊕⊕⊕⊝ Moderate	
Twetman and Dhar, 2015 [30]	Reduce the incidence of early childhood caries	The review included six trials described in seven publications assessing 5% NaF FV. Two studies were considered as of high risk of bias and the remaining were of moderate risk. The mean PF indicated in the SR was 18%, and was calculated from three studies with moderate risk of bias	⊕⊕⊝⊝ Low	There is moderate and limited quality of evidence in support of the anticaries benefit of FV in preschoolers
Mishra et al. 2017 [16]	Prevention of ECC	The review included 17 studies, the majority (*n* = 15) being RCTs and the remainder (*n* = 2) observational studies without a control group. The PF in the included studies varied from 5% to 63%. Participants had a baseline dmfs between zero and 8 in 13 included studies and the PF varied from 6.4% to 63%. In three other studies with baseline dmfs of eight to 16, the PF varied from 5% to 24.48%	⊕⊝⊝⊝ Very low	Limited quality of evidence to determine the anticaries benefit of FV in preschoolers
Sousa et al., 2019 [14]	Caries at dentine level in the primary dentition assessed by any caries index and/or measurement of disease occurrence at individual level	The proportion of children with new dentine caries lesions was reported in 16 studies involving five different comparisons. The results favoured FV in the comparisons with usual care (RR = 0.84; 95% CI 0.72, 0.98) or no intervention (RR = 0.85; 95% CI 0.73, 0.98). The other comparisons did not show this effect including the comparison between FV and placebo (RR = 0.86; 95% CI 0.72, 1.03). The remaining comparison included the one of FV, oral health advice, community health promotion and the use of 500 ppm F toothpaste versus no intervention (RR = 1.00; 95% CI 0.94, 1.06) and of FV, oral health advice, the use of 1450 ppm F toothpaste versus oral health advice (RR = 0.87; 95% CI 0.75, 1.02). The SR obtained a pooled RR of 0.88 (95% CI 0.81, 0.95), meaning an overall FV protection of 12%. The prediction interval for the pooled RR was 0.68 to 1.14. When the prediction intervals were taken into account, the results were not statistically significant for all comparisons, including the overall pooled estimate	⊕⊕⊝⊝ Low	FV made hardly any difference in the risk of developing new caries in children
	Caries at dentine level in the primary dentition assessed by any caries index and/or measurement of disease occurrence at a tooth level (dmft)	The pooled PFs for dmft data from 12 studies was 31.13% (95% CI 21.08, 41.18) and MA using the WMD for dmft resulted in pooled estimates of −0.30 (95% CI –0.69, 0.09). The PFs per comparison for dmft were as follows: 33.73 (22.78, 44.68) for FV versus placebo, 17.07 (−12.49, 38.42) for FV versus no intervention, and 28.57 (−365.42, 106.77) FV with oral health advice versus oral health advice	⊕⊕⊝⊝ Low	
	Caries at dentine level in the primary dentition assessed by any caries index and/or measurement of disease occurrence at surface level (dmfs)	The pooled PFs for dmfs data from 12 studies was 24.15% (95% CI 12.91, 35.38) and MA using the WMD for dmfs resulted in pooled estimates of −0.77 (95% CI –1.23, −0.31). The PFs per comparison for dmfs were as follows: 30.49 (8.01, 52.96) for FV versus placebo, −33.33 (−105.56, 14.52) for FV versus usual care, 23.70 (7.86, 39.55) for FV versus no intervention, and 23.96 (8.42, 37.47) for FV, oral health advice, community health promotion and the use of 500 ppm F toothpaste versus no intervention	⊕⊕⊝⊝ Low	
Yu et al., 2021 [13]	Increment of decayed (missing/extraction indicated) and filled surfaces/teeth d(m/e)fs or d(m/e)ft	d(m/e)fs increment pooled estimate of all six trials from the random‐effects MA was −0.17 (95% CI −0.60 to 0.26; *p* = 0.43), which suggests a non‐significant effect in favour of the additional use of FV. Heterogeneity of the outcome was not statistically significant	⊕⊕⊕⊝ Moderate	There is low to moderate certainty evidence that FV does not have significant additional anticaries benefits for children when used with daily tooth brushing with fluoride toothpaste (≥ 1000 ppm)
	Incidence of caries (percentage of children who developed new caries, including both those of caries‐free and already with caries at baseline)	The pooled RR (random‐effects MA) of the incidence of caries was 0.91 (95% CI 0.80 to 1.05), which suggests a non‐significant effect (*p* = 0.21) that is slightly in favour of additional use of FV. Heterogeneity was moderate in these results (*p* = 0.17; *I* ^2^ = 41%)	⊕⊕⊕⊝ Moderate	
	Changes in prevalence of caries (caries prevalence rate at follow‐up minus caries prevalence rate at baseline)	The pooled RR (random‐effects MA) of changes in prevalence of caries was 0.89 (95% CI 0.78 to 1.01; *p* = 0.07), which suggests a non‐significant effect that is slightly in favour of additional FV. Heterogeneity was not detected in these results (*p* = 0.74; *I* ^2^ = 0%)	⊕⊕⊝⊝ Low	
Manchanda et al., 2021 [20]	Caries increment in the primary dentition diagnosed visually and/or via tactile means	The effectiveness of semi‐annual application of 5% NaF varnish was evaluated in 11 studies. Six of those found a significant effect while the rest failed to demonstrate the caries protective effective of FV in preschoolers. The mean effect size of 5% NaF 6 monthly versus control was −1.56 (95% CI –2.7, −0.41). a low certainty of evidence Annual application of 5% NaF varnish was evaluated in one study. The mean effect size of 5% NaF once per year versus control was −1.53 (95% CI –3.99, −0.93). There was no effectiveness of the use of NaF varnish once a year. Certainty of evidence was moderate One trial evaluated the use of Difluorosilane varnish four times per year showing a significant result in caries prevention. The mean effect size 0.9% DFS 3 monthly versus control was −5.10 (95% CI –8.45, −1.75). Certainty of evidence was low Another trial assessed 0.9% DFS 6 monthly and failed to show the significant caries preventive benefits of the intervention. The mean effect size 0.9% DFS 6 monthly versus control was −0.10 (95% CI –2.99, −2.79). Certainty of evidence was moderate	⊕⊕⊝⊝ Low to ⊕⊕⊕⊝ Moderate	Various professionally applied FV can effectively prevent early childhood caries
Muantenu et al., 2022 [33]	Caries reduction	Among the three studies including primary teeth, anticaries agents one obtained a dEs (decayed surfaces with initial enamel lesions) of 1.20 versus 3.05 for the 5% NaF varnish versus no treatment groups. The other two studies presented PF of 28% and 49%	⊕⊝⊝⊝ Very low	FV applications are effective on preventing dental caries in primary dentition
He et al., 2023 [21]	Caries increment in the primary dentition by counting the number of new decayed teeth and tooth surfaces. Change in dmft and dmfs from baseline to the end point was defined as caries increment	The SR with NMA included 29 RCTs investigating 18 anticaries agents or their combinations. FV versus control was one of the agents investigated in more than one study, appearing in seven. FV versus control resulted in a SMD of −019 (95% CI –0.39, 0.02). According to the SUCRA results, FV plus low fluoride toothpaste and FV plus high fluoride toothpaste ranked fourth and fifth from highest to lowest probability of being the most effective in preventing caries	⊕⊝⊝⊝ Very low	There is low certainty evidence that FV is effective in reducing caries increment or caries incidence in preschool children
	Caries incidence (new caries in any tooth) at the child level	The SR with NMA included 19 RCTs investigating 18 anticaries agents or their combinations. Five included studies investigating FV versus control and resulted in an OR of 0.63 (95% CI 0.48, 0.81). FV and FV plus high fluoride toothpaste were considered effective. According to the SUCRA results, FV plus low fluoride toothpaste and FV ranked fourth and fifth from highest to lowest probability of being the most effective in preventing caries	⊕⊕⊝⊝ Low	
Rup et al., 2023 [34]			⊕⊝⊝⊝ Very low	

Abbreviations: dfs, decayed and filled primary teeth surface; dmft, decayed, missing and filled primary teeth; FV, fluoride varnish; NaF, sodium fluoride; NNT, number needed to treat; PF, prevented fraction; RCT, randomised controlled trial; SMD, standardised mean difference; SR, systematic review; SUCRA, surface under the cumulative ranking curve; WMD, weighted mean difference.

^a^ The certainty of evidence was assessed by the authors for nine systematic reviews (Bader et al., 2001 [17]; Rozier, 2001 [31]; Petersson et al., 2004 [32]; Azarpazhooh and Main, 2008 [18]; Carvalho et al., 2010 [19]; Mishra et al. 2017 [16]; Sousa et al., 2019 [14]; Muntenau et al., 2022 [33]; Rup et al.,2023 [34] while GRADE assessments reported in the reviews were used for five others) (Marinho et al., 2013 [15]; Twetman and Dhar, 2015 [30]; Yu et al., 2021 [13]; Manchanda et al., 2021 [20]; He et al., 2023 [21]).

Five SRs [14, 18, 19, 20, 33] highlighted the need for cost‐effectiveness analysis. One SR [18] descriptively presented primary studies on cost. The certainty of evidence varied from very low to moderate (Appendix S4). The RB was the criterion that most contributed to downgrading the certainty of evidence in the SRs. Many SRs [14, 15, 16, 17, 19, 20, 21, 30] made recommendations for further research, highlighting the need for better‐designed RCT (Appendix S5).

Earlier SRs have highlighted a scarcity of information regarding adverse effects [15, 16], indicating a need for further studies [18, 19]. Recent reviews that addressed adverse effects noted a low risk of allergic reactions [20], a few minor adverse effects [14], or a lack of evidence on the subject [13].

Two [31, 33] included SRs did not report any RB or MQ assessments. Seven [13, 14, 15, 20, 21, 30, 34] SRs assessed the risk of bias using the Cochrane's risk of bias tool, but one [30] did not make the assessment available. The remaining five [16, 17, 18, 19, 32], mostly earlier reviews, conducted quality assessments. One [16] used the JBI critical appraisal checklist but did not make the assessment available.

Eleven [13, 16, 17, 18, 19, 20, 30, 31, 32, 33, 34] of the 14 SR had critically low MQ (Table 3) and high RB (Table 4). The remaining three [14, 15, 21] had a low RB. One, published in 2013 and with high MQ [15], found no evidence that FV reduced the risk of developing new caries lesions. However, it did find that FV applications were associated with a substantial reduction in the dmf‐s. The two remaining SR with low RB had low MQ [14, 21]. One SR [14], published in 2019, found an overall protection of 12% at an individual level, concluding that FV made little difference in the risk of developing new caries lesions in children. The other [21], a 2023 SR with NMA, aimed to rank the effectiveness of different clinical interventions for preventing early childhood caries (ECC). It concluded that some agents, including FV, were effective in reducing caries in preschool children. However, the authors highlighted that the NMA results should be interpreted with caution due to the relatively small number of studies, confidence in the findings, and the studies' limitations.

**TABLE 3:** Quality assessment of the included SR using AMSTAR 2.

Author, year	Q1	Q2^a^	Q3	Q4^a^	Q5	Q6	Q7^a^	Q8	Q9^a^	Q10	Q11^a^	Q12	Q13^a^	Q14	Q15^a^	Q16	Overall
Bader et al., 2001 [17]	N	N	N	N	Y	Y	N	N	N	N	NMA	NMA	N	N	NMA	N	Critically low
Rozier, 2001 [31]	N	N	N	N	N	N	N	N	N	N	NMA	NMA	N	N	NMA	N	Critically low
Petersson et al., 2004 [32]	N	N	N	N	Y	Y	Y	PY	N	N	NMA	NMA	Y	N	NMA	N	Critically low
Azarpazhooh and Main, 2008 [18]	N	N	N	N	Y	N	N	Y	N	N	NMA	NMA	N	N	NMA	Y	Critically low
Carvalho et al., 2010 [19]	N	N	Y	N	Y	N	Y	PY	N	N	NMA	NMA	Y	Y	NMA	N	Critically low
Marinho et al., 2013 [15]	Y	Y	Y	Y	Y	Y	Y	Y	Y	N	Y	Y	Y	Y	Y	Y	High
Twetman and Dhar, 2015 [30]	N	N	N	N	Y	N	N	PY	N	N	NMA	NMA	Y	N	NMA	N	Critically low
Mishra et al. 2017 [16]	N	N	N	N	Y	N	N	PY	N	Y	NMA	NMA	N	N	NMA	Y	Critically low
Sousa et al., 2019 [14]	Y	N	Y	Y	N	Y	N	N	Y	N	Y	Y	Y	Y	Y	Y	Low
Yu et al.,2021 [13]	Y	N	Y	PY	Y	Y	N	PY	Y	N	Y	N	N	N	Y	Y	Critically low
Manchanda et al., 2021 [20]	Y	N	Y	PY	Y	Y	Y	PY	Y	N	N	N	N	N	Y	Y	Critically low
Muantenu et al., 2022 [33]	Y	N	Y	N	N	N	N	PY	N	N	NMA	NMA	N	N	NMA	Y	Critically low
He et al., 2023 [21]	Y	PY	N	PY	Y	Y	Y	PY	Y	N	Y	Y	Y	N	Y	Y	Low
Rup et al., 2023 [34]	Y	N	Y	PY	Y	Y	N	N	Y	N	NMA	NMA	N	N	NMA	Y	Critically low

*Note:* Questions: Q1. Did the research questions and inclusion criteria for the review include the components of PICO?; Q2. Did the report of the review contain an explicit statement that the review methods were established prior to the conduct of the review and did the report justify any significant deviations from the protocol?; Q3. Did the review authors explain their selection of the study designs for inclusion in the review?; Q4. Did the review authors use a comprehensive literature search strategy?; Q5. Did the review authors perform study selection in duplicate?; Q6. Did the review authors perform data extraction in duplicate?; Q7. Did the review authors provide a list of excluded studies and justify the exclusions?; Q8. Did the review authors describe the included studies in adequate detail?; Q9. Did the review authors use a satisfactory technique for assessing the risk of bias (RoB) in individual studies that were included in the review?; Q10. Did the review authors report on the sources of funding for the studies included in the review?; Q11. If meta‐analysis was performed, did the review authors use appropriate methods for statistical combination of results?; Q12. If meta‐analysis was performed, did the review authors assess the potential impact of RoB in individual studies on the results of the meta‐analysis or other evidence synthesis?; Q13. Did the review authors account for RoB in individual studies when interpreting/discussing the results of the review?; Q14. Did the review authors provide a satisfactory explanation for, and discussion of, any heterogeneity observed in the results of the review?; Q15. If they performed quantitative synthesis, did the review authors carry out an adequate investigation of publication bias (small‐study bias) and discuss its likely impact on the results of the review?; Q16. Did the review authors report any potential sources of conflict of interest, including any funding they received for conducting the review?

Abbreviations: N, no; NMA, no meta‐analysis conducted; PY, partial yes; Y, yes.

^a^ Items considered critical.

**TABLE 4:** Risk of bias assessment of included reviews presented according to phase two (“identify concerns with the review process”) and phase three (“judge risk of bias”) of ROBIS tool.

	Phase 2																									Phase 3
	Domain 1: study eligibility criteria						Domain 2: identification and selection of studies						Domain 3: data collection and study appraisal						Domain 4: synthesis and findings							Risk of bias in the review
	1.1	1.2	1.3	1.4	1.5	Concern	2.1	2.2	2.3	2.4	2.5	Concern	3.1	3.2	3.3	3.4	35	Concern	4.1	4.2	4.3	4.4	4.5	4.6	Concern	
Bader et al., 2001 [17]	NI	PN	N	PN	N	High	PN	PN	PN	N	Y	High	PY	N	PN	N	N	High	PY	NI	PY	PY	N	N	High	High
Rozier, 2001 [31]	NI	N	N	PY	N	High	N	N	N	N	N	High	N	N	N	N	N	High	Y	NI	N	N	N	N	High	High
Petersson et al., 2004 [32]	N	N	N	PY	N	High	N	PY	PY	N	Y	High	PY	Y	PY	PY	Y	Low	Y	NI	PY	N	N	N	High	High
Azarpazhooh and Main, 2008 [18]	NI	PY	N	PY	N	High	Y	Y	Y	N	Y	High	NI	Y	Y	N	N	High	N	NI	PN	N	N	N	High	High
Carvalho et al., 2010 [19]	NI	Y	PY	Y	N	High	PN	Y	PY	N	Y	High	NI	PY	Y	N	NI	High	Y	NI	Y	Y	PN	Y	Low	High
Marinho et al., 2013 [15]	PY	Y	Y	Y	Y	Low	Y	Y	Y	Y	Y	Low	Y	Y	Y	Y	Y	Low	Y	Y	Y	Y	PN	Y	High	Low
Twetman and Dhar, 2015 [30]	NI	Y	PY	PN	N	High	PN	Y	PY	N	Y	High	NI	Y	PN	N	PN	High	PN	NI	Y	Y	N	N	High	High
Mishra et al. 2017 [16]	NI	N	N	NI	NI	High	Y	Y	PY	PY	Y	Low	NI	PY	PN	N	N	High	PY	NI	PN	N	N	N	High	High
Sousa et al., 2019 [14]	N	PN	N	NI	NI	Unclear	Y	Y	Y	Y	NI	Unclear	Y	Y	Y	Y	Y	Low	Y	Y	Y	Y	Y	Y	Low	Low
Yu et al.,2021 [13]	Y	Y	Y	Y	Y	Low	PY	Y	Y	Y	Y	Low	Y	PN	PY	Y	Y	High	Y	N	Y	Y	PY	N	High	High
Manchanda et al., 2021 [20]	Y	Y	Y	Y	N	High	Y	PY	Y	Y	Y	Low	Y	PN	Y	Y	Y	Low	Y	PN	PY	PN	Y	N	High	High
Muantenu et al., 2022 [33]	NI	PN	N	N	N	High	PN	PN	PN	N	PN	High	PN	PY	PN	N	N	High	PN	NI	PY	N	N	N	High	High
He et al., 2023 [21]	Y	Y	Y	Y	PN	Low	PN	Y	Y	Y	Y	High	Y	PY	PY	Y	PY	Low	Y	PY	PY	PN	PY	PY	High	Low
Rup et al., 2023 [34]	Y	Y	PN	Y	Y	Low	PN	PN	Y	Y	Y	High	Y	PN	N	Y	PY	High	N	Y	N	N	PN	PY	High	High

Abbreviations: N, no; NI, no information; PN, probably no; PY, probably yes; Y, yes.

## 4 Discussion

Most of the SRs included in this overview had critically low MQ and a high RB. Seven SRs [15, 18, 20, 21, 30, 33, 34] found FV effective, five SRs [16, 17, 19, 31, 32] reported insufficient evidence to draw reliable conclusions, and two SRs questioned the clinical significance of FV applications [13, 14].

A SR is only as up‐to‐date as the date of its literature search, making it susceptible to becoming outdated [39]. High MQ alone does not ensure a better evidence base if recent studies are excluded. The only SR of high MQ lacks recent updates [15], and since its publication, new RCTs on the effectiveness of FV have emerged. Systematic Reviews should be periodically updated, with Cochrane reviews considered trustworthy; hence, it is surprising that the last update to the Cochrane review on FV occurred in 2013 [15], especially since more recent well‐conducted studies have found little to no anticaries effect of FV [8, 9, 11, 12, 40, 41].

The certainty of evidence as assessed in the 2013 Cochrane review [15] may be questioned. While the authors downgraded the certainty of evidence due to high heterogeneity and RB, they upgraded it based on a consistent and large clinically important effect. Although a large magnitude of effect can justify upgrading certainty from strong observational studies [27], the FV effect was derived from RCTs. Among the 10 trials on primary dentition included in the review, half were at high RB, and the other half had an unclear risk. Since trials with a high RB tend to overestimate the effect of interventions [42], upgrading the certainty of evidence for the anticaries effect of FV from low to moderate is questionable. Claiming that the effectiveness of FV is substantial—based on a questionable assessment of the certainty of evidence as moderatemay mislead readers who only read the conclusion, as it could make the results appear more favourable than the data actually support.

Five included SRs [14, 18, 19, 20, 33] mentioned cost‐effectiveness. One SR [18] suggested FV is cost‐effective in preventing dental caries; however, the supporting evidence is weak. This SR [18] cited an outdated study [43] that cannot be considered a full economic evaluation of the cost‐effectiveness type, as it did not compare two interventions to consider both costs and outcomes [44],and a study [45] in which FV was not cost‐saving in the first 42 months of life, and did not conclude that FV is cost‐effective. This limits the ability to draw reliable conclusions about the potential cost‐effectiveness of FV from these data. The other four SRs [14, 19, 20, 33] suggested conducting studies to assess FV's cost‐effectiveness. A recent SR [46] complied with this recommendation and assessed cost‐effectiveness as the primary outcome and found no convincing evidence that FV is cost‐effective in preventing caries in preschoolers.

Methodological quality and risk of bias may seem inversely related, but high MQ of a SR does not necessarily imply a low RB, and vice versa. This discrepancy may arise because some evaluation criteria differ between these assessments; the differences can stem from how criteria are evaluated or from criteria being included in only one of the assessments. Among the 11 SRs with a high RB, all were found to have critically low quality. However, of the three SRs with a low RB, only the Cochrane SR [15] had high MQ. The remaining two SRs [14, 21], despite their low RB, were identified as having low MQ.

Sousa et al. [14] reported the number of studies excluded for each reason, but did not provide a list of potentially relevant studies excluded after full‐text review. The absence of this information reduced the quality of the SR to low, but did not affect the RB. In He et al. [21], the AMSTAR‐2 critical item concerning the search strategy received a “partial yes” judgement due to the exclusion of study registries or grey literature. Since AMSTAR‐2 does not specify how to handle “partial yes” evaluations, this was treated as “no,” leading to a downgrade to low MQ. Similarly, this SR received a “probably not” for the search strategy in phase two of the ROBIS assessment. It followed the ROBIS guideline, which recommends including at least MEDLINE and EMBASE databases, but the authors acknowledged the limitation of their search. The overall assessment in phase three resulted in a low RB.

Three SRs had low RB [14, 15, 21]; however, one needs updating [15], while the other two have low certainty of evidence [14, 21]. These SRs differ on whether FV effectively reduces caries or makes little difference. Nine RCTs published since the latest version of the Cochrane review [15] are included in these two most recent reviews with low RB [14, 21]. Among these RCT, one [47] reported that oral hygiene instruction combined with FV and casein phosphopeptide–amorphous calcium phosphate (CPP‐ACP) mousse reduced the size of white spot lesions and decreased the dmft index in primary teeth. The remaining eight studies [8, 9, 10, 11, 12, 40, 48, 49] found no anti‐caries benefit of FV in preschoolers.

The limited additional benefit [13] and relatively little clinical relevance [14] of FV suggest that applying it to all children may be unnecessary; still, several guidelines recommend its use for caries prevention in all preschoolers [7]. Some guidelines advise applying FV for all children but adjusting the frequency based on caries risk [7]. However, determining caries risk is challenging—while caries history is the best single indicator, multivariate models appear to provide better but still relatively inaccurate predictions [50]. Although FV is typically recommended for children at high risk of caries [13, 14, 16, 17, 18], its anticaries benefit remains relatively limited even in this group, offering little justification for widespread use.

There were some deviations from protocol. This overview was intended to follow the Preferred Reporting Items for Systematic Reviews and Meta‐Analyses (PRISMA 2020) [51] with necessary adaptations for an overview. However, since the protocol was registered, a new reporting guideline for overviews, the PRIOR statement [23], was published and was subsequently used for reporting this overview. The protocol originally specified the use of the GRADE approach for extracting and evaluating the quality or certainty of evidence. However, since SR with NMA were also included, the CINeMA assessment [29] was used to collect the quality or certainty of evidence in these SRs.

This study has limitations, such as focusing solely on published SRs and not including a search of the grey literature, which could potentially uncover additional studies. Additionally, the certainty of evidence was assessed by the authors only when not provided in the SR. Although a systematic approach was employed for this evaluation, the subjective nature of such assessments could lead to different ratings if conducted by other investigators.

Despite the large number of publications, only a few SRs have a low risk of bias, and the certainty of the evidence regarding the effectiveness of FV remains low. New, pragmatic, multi‐center, and higher‐quality RCTs could strengthen the evidence and address unresolved questions. More recent RCTs [8, 9, 10, 11, 12] suggest no or a limited benefit from FV application in both low‐ and high‐risk children [9, 11, 12]. To more clearly determine whether there is a difference in the anticaries benefit of FV for children at varying caries risks, a RCT with stratified randomization, including both low‐ and high‐risk children in the intervention and control groups, is recommended. Another question for future RCTs is whether more frequent applications, such as every 3 months, confer greater benefit. These trials should be incorporated into well‐conducted SRs that assess not only the statistical significance of the intervention but also its clinical relevance.

Many systematic reviews have assessed the effectiveness of fluoride varnish in preventing caries in preschoolers, but most are of low quality, with a high risk of bias and conflicting findings. Based on updated, lower‐risk reviews [14, 21] that included the latest trials, fluoride varnish offers limited additional benefit to children already receiving optimal fluoride exposure through fluoridated toothpaste and water. Therefore, the routine application of fluoride varnish should be reconsidered.

## Author Contributions

Flávia Macedo Couto, Fernanda Santos de Oliveira de Sousa, Fernanda Barja‐Fidalgo, Ana Paula Pires dos Santos, and Paulo Nadanovsky contributed to the conception and design of the work as well as the acquisition, analysis, interpretation of data, and drafting the manuscript. Izabel Monteiro Dhyppolito contributed to the conception and design of the work as well as drafting the manuscript. All authors critically revised the paper for important intellectual content; gave final approval of the version to be published; and agreed to be accountable for all aspects of the work in ensuring that questions related to the accuracy or integrity of any part of the work are appropriately investigated and resolved.

## Conflicts of Interest

The authors declare no conflicts of interest.

## Supporting information


**Appendix S1:** cdoe70032‐sup‐0001‐AppendixS1.docx.

**Appendix S2:** cdoe70032‐sup‐0002‐AppendixS2.docx.

**Appendix S3:** cdoe70032‐sup‐0003‐AppendixS3.docx.

**Appendix S4:** cdoe70032‐sup‐0004‐AppendixS4.docx.

**Appendix S5:** cdoe70032‐sup‐0005‐AppendixS5.docx.

## Data Availability

The collected data that support the findings of this study will be openly available in OSF.IO at https://osf.io/kc2xn/; https://doi.org/10.17605/OSF.IO/KC2XN.
