# Supplementary material for: Fluoride Varnish for Caries Prevention in Preschoolers: An Overview of Reviews
Source: Community Dent Oral Epidemiol. 2025 Nov 20;54(2):203–19. doi: 10.1111/cdoe.70032 (PMC13000968; doi:10.1111/cdoe.70032)
Supplement: Supplementary file 1 — Appendix S1: cdoe70032‐sup‐0001‐AppendixS1.docx. [file CDOE-54-203-s004.docx]

**Appendix 1 - Search strategy**

**Pubmed**

("fluoride varnish" OR AllSolutions Fluoride Varnish [Supplementary Concept]) AND systematic [sb]

**Epistemonikos**

(title:("fluoride varnish") OR abstract:("fluoride varnish")) AND (title:(AllSolutions Fluoride Varnish [Supplementary Concept]) OR abstract:(AllSolutions Fluoride Varnish [Supplementary Concept])) OR (title:("systematic review") OR abstract:("systematic review")) OR (title:(systematic*) OR abstract:(systematic*)) AND (title:(review) OR abstract:(review)) OR (title:(Systematic Reviews as Topic) OR abstract:(Systematic Reviews as Topic)) OR (title:(Systematic Review [Publication Type]) OR abstract:(Systematic Review [Publication Type])) OR (title:(meta-analysis) OR abstract:(meta-analysis)) OR (title:(meta-analysis) OR abstract:(meta-analysis)) OR (title:((Meta-Analysis [Publication Type])) OR abstract:((Meta-Analysis [Publication Type]))) OR (title:(Meta-Analysis as Topic) OR abstract:(Meta-Analysis as Topic))

**Scopus**

"fluoride varnish" AND systematic AND review OR meta-analysis

**Web of Science**

"fluoride varnish" AND (("systematic review" OR (systematic* AND review) OR Systematic Reviews as Topic OR Systematic Review [Publication Type] OR meta-analysis OR meta-analysis OR (Meta-Analysis [Publication Type]) OR (Meta-Analysis as Topic)))

**EMBASE**

'fluoride varnish' AND ('systematic review' or 'meta analysis')

**Cochrane**

*fluoride varnish

**Health Evidence**

("fluoride varnish" OR AllSolutions Fluoride Varnish [Supplementary Concept]) AND (("systematic review" OR (systematic* AND review) OR Systematic Reviews as Topic OR Systematic Review [Publication Type] OR meta-analysis OR meta-analysis OR (Meta-Analysis [Publication Type]) OR (Meta-Analysis as Topic)))

**BVS**

fluoride varnish AND Systematic Review or Meta-analysis
