# Supplementary material for: Fluoride Varnish for Caries Prevention in Preschoolers: An Overview of Reviews
Source: Community Dent Oral Epidemiol. 2025 Nov 20;54(2):203–19. doi: 10.1111/cdoe.70032 (PMC13000968; doi:10.1111/cdoe.70032)
Supplement: Supplementary file 2 — Appendix S2: cdoe70032‐sup‐0002‐AppendixS2.docx. [file CDOE-54-203-s005.docx]

**Appendix 2 -** Characteristics of the included Systematic Reviews

| **OVERVIEW OF REVIEWS EVALUATING THE EFFECT OF PROFESSIONAL FLUORIDE VARNISH APPLICATIONS ON THE INCIDENCE OF DENTAL CARIES IN PRESCHOOLERS** | |
| --- | --- |
| **Bader_2001**  Title: A systematic review of selected caries prevention and management methods  Metanalysis: No meta-analysis conducted  Authors: James D. Bader, Daniel A. Shugars and Arthur J. Bonito  Reference and language of publication: A systematic review of selected caries prevention and management methods. Community Dent Oral Epidemiol 2001; 29: 399–411. ENG.  Corresponding author's contact: jim_bader@unc.edu  Information collected by: Flávia  Date: **16/12/2023** | |
| **Review Methods** | |
| 1. Aim | To determine the strength of the evidence for the efficacy of professional caries preventive methods applied to high risk individuals, and the efficacy of professionally applied methods to arrest or reverse non-cavitated carious lesions |
| 2. Inclusion criteria | we limited studies to in vivo designs involving human subjects  we included reports involving preventive or management interventions requiring professional application or prescription, or interventions likely to be undertaken only upon the recommendation of a dentist.  Because we expected some findings to be subgroup analyses, we did not establish a minimum sample size.  For the question involving the efficacy of preventive interventions in caries-active or high risk groups We accepted caries-active or high caries risk classifications based on any combination of decayed, filled and/or missing primary and/or permanent surface or tooth scores. We also included all studies where risk was established through microbiological  testing. |
| 3. Exclusion criteria | Excluding in situ and in vitro studies.  We excluded all studies without concurrent control groups (nil, placebo, or active)  We specifically excluded only dentifrice studies where dentifrice use was not a part of a larger intervention.  For the question involving the efficacy of preventive interventions in caries-active or high risk groups, we excluded studies where such classifications were not made on an individual basis. We excluded studies where outcomes were not expressed in terms of numbers of decayed and filled, or decayed, missing and filled surfaces or teeth. |
| 4. Bases Searched and limitations (date and language) | we conducted a detailed search of the relevant English language literature from 1966 to October of 1999 using MEDLINE, EMBASE and the Cochrane controlled trials register.  We did not pursue reports in the gray literature, defined as theses, dissertations, product reports and unpublished studies.  We did hand search the most fruitful journals from 1998 to the end of 1999 to accommodate for the lag in MEDLINE postings. The search focused on dental caries preventive or management methods, using keywords for methods (fluorides, topical; fluoride supplements; pit and fissure sealants; health education, dental; dental prophylaxis; oral hygiene; dental plaque; chlorhexidine; xylitol; cariostatic agents) and study design, in addition to the disease key words. |
| 5. PRISMA diagram | Not mentioned |
| 6. Population | Caries active/high risk individuals |
| 7. Intervention | Fluorides (professional methods) |
| 8. Comparators | nil, placebo, or active |
| 10. Primary and secondary outcomes | incidence of new coronal carious lesions in primary and permanent teeth / numbers of decayed and filled, or decayed, missing and filled surfaces or teeth. |
| 11. Quality and bias assessment method | We also computed a quality score for each included study using a quality rating form covering several elements of internal validity. The items and proportion of the overall score weight they represented addressed duration (15%), sample size (15%), study type (10%), blinding (10%), examiner reliability (10%), baseline assessments of differences among groups (5%), loss to follow-up (5%), previous/concurrent prevention exposure (5%), intention-to-treat analyses (5%), criteria for non-cavitated lesions (caries management study only) or proportion of population designated as high-risk (prevention study only) (5%) and reviewer’s subjective assessment of both internal (7.5%) and external validity (7.5%) of  the study. For these latter two items, reviewers were directed to consider whether one or more threats to internal validity were present, and whether the results could be generalized beyond small, very specific populations. For most studies, quality scores could range from 0 to 20, although for some studies not all items were appropriate, and maxima of 18 and 19 were used. All scores were rescaled to a 0–100 scale. |
| **Results** | |
| 12. Number of included studies and participants | Nine evaluations reported in seven studies examined the efficacy of fluorides for the  prevention of carious lesions. All of the studies involved children as subjects; one study examined effects on primary teeth (18).  Five of the interventions involved the use of fluoride varnish. |
| 13. SRs assessment of the certainty of evidence | We judged the evidence for efficacy to be fair for fluoride varnishes and insufficient for other fluoride based methods, based primarily on small numbers of studies of any type of intervention upon which to determine efficacy.  Fair: Data are sufficient for evaluating efficacy. The sample size is adequate, but the data show some inconsistencies in outcomes between intervention and placebo/usual care groups such that efficacy is not clearly established. |
| 14. Reporting on limitations | The principal shortcomings are the number of available studies for any given intervention, the variety of experimental protocols among any set of studies, the lack of studies including adult subjects and root surfaces, the meager number of studies examining effects on primary teeth, the identification of caries-active and at risk subjects, and several study design issues.  Nevertheless, the small number of available studies limits conclusions that can be drawn  about the efficacy of any specific preventive approach among caries-active/high risk individuals.  Our ability of draw conclusions about any specific preventive approach is further limited by the variation in experimental protocols. For example, fluoride varnish was one of the few interventions for which we were able to rate the evidence at a level other than ‘‘incomplete.’’ Yet, among five fluoride varnish studies, three different concentrations of two different varnishes were evaluated using three different application frequencies. Comparison groups in these five studies received three different types of treatment, and both experimental and comparison subjects received five different patterns of additional community and individual preventive procedures during the course of the trials. |
| 15. Reporting on recommendations | Not mentioned |

| **OVERVIEW OF REVIEWS EVALUATING THE EFFECT OF PROFESSIONAL FLUORIDE VARNISH APPLICATIONS ON THE INCIDENCE OF DENTAL CARIES IN PRESCHOOLERS** | |
| --- | --- |
| **ROZIER_2001**  Title: Effectiveness of Methods Used by Dental Professionals for the Primary Prevention of Dental Caries  Metanalysis: NO  Authors: R. Gary Rozier  Reference and language of publication: Rozier RG. Effectiveness of methods used by dental professionals for the primary prevention of dental caries. J Dent Educ. 2001 Oct;65(10):1063-72. PMID: 11699978. ENG.  Corresponding author's contact: gary_rozier@unc.edu  Information collected by: Fernanda Sousa  Date: 20/12/2023 | |
|  | |
| **Review Methods** | |
| 1. Aim | the paper seeks to answer the following question for each of the preventive methods: “Overall, how effective is this agent or activity when used in a typical dental office setting with a typical patient?”  A systematic review was also done on the effectiveness of fluoride varnish in inhibiting caries in primary teeth. |
| 2. Inclusion criteria | All fluoride varnish papers retrieved from MEDLINE for 1966-2000 using the keywords  “topical fluoride” were reviewed and entered into evidence tables if they included assessments of primary tooth caries increments in experimental and control groups. |
| 3. Exclusion criteria | Not mentioned |
| 4. Information sources (date and language) | from MEDLINE for 1966-2000 using the keywords “topical fluoride”  papers published in the English language |
| 5. PRISMA diagram | Not available |
| 6. Population | Preschool-aged children |
| 7. Intervention | Fluoride varnish |
| 8. Comparators | Not mentioned |
| 9. Primary and secondary outcomes | Two measures of treatment effect are presented in summary tables. The primary measure of outcome is the Prevented Fraction (PF), or the proportional reduction in dental caries between experimental and control participants, expressed as a percentage |
| 10. Quality and risk of bias assessment method | scored for quality using the rating forms devised by the Research Triangle Institute-University of North Carolina Evidence-Based Practice Center for its review of management of dental  Caries The overall quality score, which can vary from 0 to 100, is based on several items of internal validity and a subjective assessment of internal and external validity |
| **Results** | |
| 11. Number of included studies and participants | Seven studies of the effectiveness of fluoride varnish when used in primary teeth were found. |
| 12. Assessment of the certainty of evidence | The strength of the evidence of effectiveness for each study was judged as “good,” “fair,” “poor,” or “insufficient” according to criteria specified by the Research Triangle Institute-University of North Carolina Evidence-Based Practice Center. |
| 13. Reporting on limitations | this review does not include use of combinations of professionally applied preventive methods |
| 14. Reporting on recommendations | the evidence of effectiveness for fluoride varnish is insufficient to recommend for or against its use in the preschool-aged child. |

| **OVERVIEW OF REVIEWS EVALUATING THE EFFECT OF PROFESSIONAL FLUORIDE VARNISH APPLICATIONS ON THE INCIDENCE OF DENTAL CARIES IN PRESCHOOLERS** | |
| --- | --- |
| **Petersson_2004**  Title: Professional fluoride varnish treatment for caries control: a systematic review of clinical trials  Metanalysis: No metanalysis  Authors: Lars G Petersson, Svante Twetman, Helena Dahlgren, Anders Norlund, Anna-Karin Holm, Gunilla Nordenram, Folke Lagerlöf, Birgitta Söder, Carina Källestål, Ingegerd Mejàre, Susanna Axelsson, Peter Lingström (12)  Reference and language of publication: Acta Odontol Scand . 2004 Jun;62(3):170-6. doi: 10.1080/00016350410006392. English  Corresponding author's contact: lars.g.petersson@lthalland.se  Information collected by: Flávia  Date: **14/12/2023** | |
| **Review Methods** | |
| 1. Aim | To report the findings concerning the caries preventive effect of topical fluoride varnish applications applied by professionals in patients of various ages |
| 2. Inclusion criteria | Randomized controlled trial of at least 2 years and with coronal caries increment in the permanent and deciduous dentition as outcome measures. Only original papers. Swedish, Danish, Norwegian, English, German, French, Italian and Spanish |
| 3. Exclusion criteria | Double publications, interim reports, abstracts, letters, short communications and chapters in textbooks. Studies using split-mouth |
| 4. Bases Searched and limitations (date and language) | Medline e Cochrane library from 1966 to april 2003 |
| 5. PRISMA diagram | Not mentioned |
| 6. Population | Not mentioned |
| 7. Intervention | Fluoride varnish |
| 8. Comparators | Placebo, no active treatment, or other fluoride preventive regimens |
| 10. Primary and secondary outcomes | coronal caries increment in the permanent and deciduous dentition |
| 11. Quality and bias assessment method | Predetermined criteria for methodology and performance |
| **Results** | |
| 12. Number of included studies and participants | 24 studies, 1200 children |
| 13. SRs assessment of the certainty of evidence | According to the protocol of the Swedish council on technology assessment in health care  Evidence was inconclusive in primary dentitio |
| 14. Reporting on limitations | No report |
| 15. Reporting on recommendations | No report |

| **OVERVIEW OF REVIEWS EVALUATING THE EFFECT OF PROFESSIONAL FLUORIDE VARNISH APPLICATIONS ON THE INCIDENCE OF DENTAL CARIES IN PRESCHOOLERS** | |
| --- | --- |
| **AZARPAZHOOH_2008**  Title: Fluoride Varnish in the Prevention of Dental Caries in Children and Adolescents: A Systematic Review  Metanalysis: No  Authors: Amir Azarpazhooh, Patricia A. Main  Reference and language of publication: Journal of the Canadian Dental Association. February 2008, Vol. 74, No. 1  Corresponding author's contact: amir.azarpazhooh@dentistry.utoronto.ca  Information collected by: Fernanda Sousa  Date: 14/12/23 | |
|  | |
| **Review Methods** | |
| 1. Aim | To develop a scientifically current and evidence-based protocol for the use  of fluoride varnish for the prevention of dental caries among high-risk children and  adolescents. More specifically, the authors of the report attempted to answer the following questions:   1. How effective is fluoride varnish in preventing dental caries in a predominantly high-risk population? In particular, how effective is fluoride varnish for young children? |
| 2. Inclusion criteria | Original research articles addressing the efficacy of varnish, protocols for its use or its toxic effects, as well as review articles providing background information, were included.  The searches were limited to articles in English and those concerning humans. Other inclusion criteria were age 0–18 years and year of publication from 2000 to 2007.  Only studies with a score of at least 11 (out of a maximum score of 16) using “Checklist to Assess Evidence of Efficacy of Therapy or Prevention” were included as the evidence for this review (n = 7). |
| 3. Exclusion criteria | Articles that did not concern the efficacy of varnish, protocols for the use of varnish or the toxic effects of varnish or that did not provide background information (review articles or guidelines) were excluded. |
| 4. Information sources (date and language) | Ovid MEDLINE (In-Process and Other Non-Indexed Citations, Daily Update), CINAHL  (Cumulative Index to Nursing and Allied Health Literature), the Evidence Based Medicine section of the Cochrane Central Register of Controlled Trials, the Cochrane Database of Systematic Reviews, the Database of Abstracts of Reviews of Effects, EMBASE, Health and Psychosocial Instruments, HealthSTAR, International Pharmaceutical Abstracts, Journals@Ovid and *ACP Journal Club*.  Period from 2000 to 2007  Review articles and all known guidelines were retrieved and reviewed for their conclusions and to identify additional citations. |
| 5. PRISMA diagram | Not available |
| 6. Population | Children and adolescents |
| 7. Intervention | Use of fluoride varnish |
| 8. Comparators | Not mentioned |
| 9. Primary and secondary outcomes | the efficacy of varnish, protocols for its use or its toxic effects |
| 10. Quality and risk of bias assessment method | The strength and quality of each study were determined according to the evidence classification system developed by the Canadian Task Force on Preventive Health Care. This system includes a hierarchy of evidence, from the highest (level I; properly randomized controlled trials) to the lowest (level III; opinions of respected authorities, based on clinical experience, descriptive studies or reports of expert committees). |
| **Results** | |
| 11. Number of included studies and participants | The evidence from these 7 studies, which was deemed to represent the best available evidence, was summarized according to the inclusion criteria. Total number of participants was not available. |
| 12. Assessment of the certainty of evidence | Canadian Task Force on Preventive Health Care. This system includes a hierarchy of evidence, from the highest (level I; properly randomized controlled trials) to the lowest (level III; opinions of respected authorities, based on clinical experience, descriptive studies or reports of expert committees). |
| 13. Reporting on limitations | No reporting on limitations |
| 14. Reporting on recommendations | For predominantly high-risk populations (e.g., people  with low socioeconomic status, new immigrants  and refugees, all First Nations and Inuit children  and adolescents), fluoride varnish should be applied  twice a year, unless the individual has no risk of caries,  as indicated by past and current caries history. |

| **OVERVIEW OF REVIEWS EVALUATING THE EFFECT OF PROFESSIONAL FLUORIDE VARNISH APPLICATIONS ON THE INCIDENCE OF DENTAL CARIES IN PRESCHOOLERS** | |
| --- | --- |
| **CARVALHO, D.M._2010**  Title: Fluoride varnishes and caries incidence decrease in preschool children: a systematic review  Metanalysis: NO  Authors: Denise Martins Carvalho, Mariana Salazar, Branca Heloísa de Oliveira, Evandro Silva Freire Coutinho  Reference and language of publication: Rev Bras Epidemiol 2010; 13(1): 1-11 – English  Corresponding author's contact: denise_dentist@yahoo.com.br  Information collected by: Fernanda Sousa  Date: 11/12/2023 | |
|  | |
| **Review Methods** | |
| 1. Aim | The objective of this systematic review is to assess whether there is evidence that professional application of fluoride varnish reduces the incidence of dental caries in primary dentition in children of up to six years of age. |
| 2. Inclusion criteria | Type of study: randomized, controlled clinical trials or quasi-randomized stu­dies.  · Type of population: children of up to six years of age, regardless of their caries ex­perience at the start of the study (initial dmfs ≥ 0).  · Type of intervention: application of topical fluoride in the form of a varnish, to primary dentition, in any quantity, concentration or application interval, on surfaces selected or not, using any application technique.  · Outcome: incidence of caries, given the presence of a cavitated lesion (level of detection C2 - enamel caries, or C3 - dentine caries) in primary dentition (dmfs).  · Languages: English, Spanish or Portu­guese; |
| 3. Exclusion criteria | Publications excluded were those that used the split-mouth design and intentio­nally administered other fluoride products, in addition to the varnish, to the test or control group (unequal co-intervention). |
| 4. Information sources (date and language) | Bibliographic searches were conducted of the BBO and LILACS databases using the terms “fluoride varnish”, “barnices flu­orados” and “verniz fluoretado”. The terms “fluoride varnish” and “dental caries” were used on the Medline database and, in order to increase the specificity of the search, the filters “humans” and “all child” were added. Next, a search was performed of the Cochra­ne Library, using the expressions “fluoride varnish”, “dental caries” and “child”. We sought to identify all articles related to the topic published up to December 2008. |
| 5. PRISMA diagram |  |
| 6. Population | children of up to six years of age, regardless of their caries ex­perience at the start of the study (initial dmfs ≥ 0) |
| 7. Intervention | application of topical fluoride in the form of a varnish, to primary dentition, in any quantity, concentration or application interval, on surfaces selected or not, using any application technique |
| 8. Comparators |  |
| 9. Primary and secondary outcomes | incidence of caries, given the presence of a cavitated lesion (level of detection C2 - enamel caries, or C3 - dentine caries) in primary dentition (dmfs). |
| 10. Quality and risk of bias assessment method | Jadad’s scale8 was used for qualitative ranking of the publications. This instrument was used to assign ratings to the studies, which varied from zero to five, based on the following criteria: method of randomi­zation, method of blinding and description of withdrawals and dropouts. |
| **Results** | |
| 11. Number of included studies and participants | only eight articles were selected for inclusion in this review.  In these clinical trials, 2,501 children, aged six months to five years, were assigned to the test or control group. |
| 12. Assessment of the certainty of evidence | Not declared |
| 13. Reporting on limitations | The problems associated with the design of the clinical studies included in this review should also be taken into consideration. We also found im­pediments to performing metanalysis, such as asymmetry and the lack of information on standard deviations.  Examination of Table 2 suggests a major asymmetry in the distribution of the data on the mean number of carious, missing and filled dental surfaces (dmfs). Several means showed values less than twice the standard deviation17, which ruled out the calculation of combined means using the weighted mean difference and the standar­dized mean difference. It was not possible to perform transformations to make the data more symmetrical since the raw data from the studies was not available. For the same reason, meta-regression was not used to assess sources of heterogeneity between the studies. |
| 14. Reporting on recommendations | It is recommended that well-designed, randomized clinical trials be con­ducted along this line of investigation. |

| **OVERVIEW OF REVIEWS EVALUATING THE EFFECT OF PROFESSIONAL FLUORIDE VARNISH APPLICATIONS ON THE INCIDENCE OF DENTAL CARIES IN PRESCHOOLERS** | |
| --- | --- |
| **MARINHO_2013**  Title: Fluoride varnishes for preventing dental caries in children and adolescents  Metanalysis: YES  Authors: Marinho VCC, Worthington HV, Walsh T, Clarkson JE  Reference and language of publication: Marinho VCC, Worthington HV, Walsh T, Clarkson JE. Fluoride varnishes for preventing dental caries in children and adolescents. Cochrane Database of Systematic Reviews 2013, Issue 7. Art.No.: CD002279. DOI: 10.1002/14651858.CD002279.pub2.  Corresponding author's contact: Helen V Worthington, Cochrane Oral Health Group, School of Dentistry, The University of Manchester, Coupland III Building, Oxford Road, Manchester, M13 9PL, UK. helen.worthington@manchester.ac.uk  Information collected by: Fernanda Sousa  Date: 15/12/2023  This review updates a previous article first published in 2002.  Protocol first published: Issue 3, 2000. (NO LONGER AVAILABLE) | |
|  | |
| **Review Methods** | |
| 1. Aim | To determine the effectiveness and safety of fluoride varnishes in preventing dental caries in the child/adolescent population. |
| 2. Inclusion criteria | Randomised or quasi-randomised controlled trials using or indicating blind outcome assessment, in which fluoride varnish is compared concurrently to a placebo or no treatment group during at least one year. |
| 3. Exclusion criteria | We excluded randomised or quasi-randomised controlled trials using within-group paired comparison designs (e.g. split-mouth trials), or with open outcome assessment or no indication of blind outcome assessment, or lasting less than one year, or controlled trials where random or quasi-random allocation was not used or indicated. |
| 4. Information sources (date and language) | We searched the following electronic databases: • The Cochrane Oral Health Group’s Trials Register; CENTRAL (The Cochrane Library); • MEDLINE via OVID; • EMBASE via OVID; • CINAHL via EBSCO; • LILACs via BIREME Virtual Health Library; • BBO via BIREME Virtual Health Library; • ProQuest Dissertations and Theses; • Web of Science Conference Proceedings.  No restrictions were placed on language or date of publication in the search of the electronic databases.  A search of the National Institutes of Health registry and results service (ClinicalTrials.gov) was undertaken on 13 May 2013  All eligible trial reports, previous meta-analyses and review articles were scanned for relevant references.  For the update of this review, only handsearching done as part of the Cochrane Worldwide Handsearching Programme was carried out.  For the original review, we contacted experts in the field of preventive dentistry to identify any unpublished trials or trials which may not be indexed by the major databases.  A list of manufacturers of fluoride varnishes was created for locating unpublished trials, and three fluoride varnish manufacturers were contacted in October 2000 and in December 2012. |
| 5. PRISMA diagram |  |
| 6. Population | Children or adolescents aged 16 or less at the start of the study (irrespective of initial level of dental caries, background exposure to fluorides, dental treatment level, nationality, setting where intervention is received or time when it started). |
| 7. Intervention | Topical fluoride in the form of varnishes only, using any fluoride agent, at any concentration (ppm F), amount or duration of application, and with any technique of application, prior or post application. However, frequency of application should have been at least once a year. |
| 8. Comparators | The control group is placebo or no treatment |
| 9. Primary and secondary outcomes | The primary outcome measure in this review was caries increment, as measured by change from baseline in the number of decayed, (missing) and filled permanent surfaces / number of decayed, (extracted/missing) and filled primary surfaces (D(M)FS / d(e/m)fs). Caries is defined here as being recorded at the dentine level of diagnosis.  The following outcomes were considered relevant: coronal dental caries and dental fillings, in both the permanent and the primary dentitions, tooth loss, dental pain, specific adverse effects (oral allergic reactions, mucosal irritation, adverse symptoms such as nausea, gagging, vomiting), use of health service resources (such as visits to dental care units, length of dental treatment time). |
| 10. Quality and risk of bias assessment method | At least two review authors undertook the assessment of the risk of bias in all of the included trials independently. This was carried out using The Cochrane Collaboration’s tool for assessing risk of bias as outlined in the Cochrane Handbook for Systematic Reviews of Interventions version 5.1 (Higgins 2011), but according to pre-defined criteria which were adapted and refined for the Cochrane topical fluoride reviews updates. |
| **Results** | |
| 11. Number of included studies and participants | Twenty-two trials with 12,455 participants randomised (9595 used in analyses) were included. |
| 12. Assessment of the certainty of evidence | Deciduous tooth surfaces d((e)/mfs increment PF- nearest to 3 years (10 trials, 3804 participants). The quality of the evidence was downgraded due to considerable heterogeneity; 5 trials were at high and 5 trials at unclear risk of bias.  However, this body of evidence showed a consistent, large clinically important effect and we have upgraded the quality of evidence to moderate.  **Moderate quality:** Further research is likely to have an important impact on our confidence in the estimate of effect and may change the estimate. |
| 13. Reporting on limitations | Yes |
| 14. Reporting on recommendations | **Implications for practice**  This review has found that the application of fluoride varnishes two to four times a year, either in the permanent or primary dentition, is associated with a substantial reduction in caries increment. |

| **OVERVIEW OF REVIEWS EVALUATING THE EFFECT OF PROFESSIONAL FLUORIDE VARNISH APPLICATIONS ON THE INCIDENCE OF DENTAL CARIES IN PRESCHOOLERS** | | | |
| --- | --- | --- | --- |
| **Twetman_2015**  Title: Evidence of Effectiveness of Current Therapies to Prevent and Treat Early Childhood Caries  Metanalysis:  Authors: Svante Twetman, Vineet Dhar  Reference and language of publication: Pediatr Dent . May-Jun 2015;37(3):246-53.  Corresponding author's contact: stwe@sund.ku.dk  Information collected by: Flávia  Date: **16/12/23** | | | |
| **Review Methods** | | | |
| 1. Aim | | (1) Do self-applied and professionally applied fluorides reduce the incidence of early childhood caries? | |
| 2. Inclusion criteria | | For the prevention sections (questions one and two), only prospective, randomized, and non-randomized controlled trials describing a defined intervention implemented to children before three years of age were considered. Furthermore, an endpoint reporting caries prevalence and/or incidence over a study period of at least one year was required. | |
| 3. Exclusion criteria | | Studies reporting surrogate endpoints or interventions directed to mothers (primary-primary prevention) were excluded. | |
| 4. Bases Searched and limitations (date and language) | | A broad search for articles published in English was conducted in the PubMed database and Cochrane library. The main search terms, in various combinations, were: early childhood caries; nursing caries; infant caries; prevention; fluoride; fluoride varnish; antibacterial agents; caries control; caries management; and restorative treatment. Relevant papers published between 2007 and April 2014 (prevention of ECC) were identified after an independent review of the abstracts by the authors (Figure 1). Diverging opinions were resolved in consensus. Reference lists of accepted papers and  systematic reviews were hand-searched for additional literature. | |
| 5. PRISMA diagram | | No PRISMA diagram, but has a flowchart  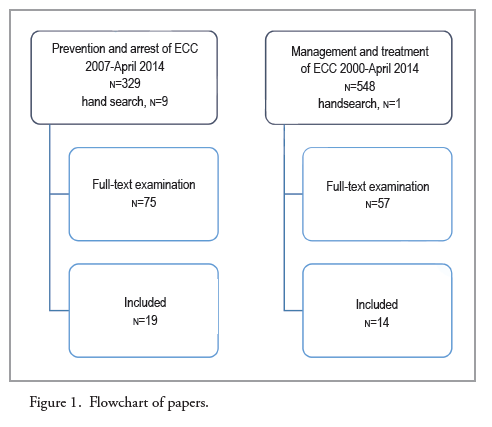 | |
| 6. Population | | Children before three years | |
| 7. Intervention | | professionally applied fluoride varnish | |
| 8. Comparators | | Not mentioned | |
| 10. Primary and secondary outcomes | | reduce the incidence of early childhood caries? caries prevalence and/or incidence over a study period of at least one year | |
| 11. Quality and bias assessment method | | The criteria of the Cochrane handbook for interventions were used, and the risk of bias for each paper was graded as low, moderate, or high. | |
| **Results** | | | |
| 12. Number of included studies and participants | | Seven papers describing six studies with fluoride varnish (five percent sodium fluoride) applications, typically two to four times per year, in combination with oral health promotion were included and 3485 children | |
| 13. SRs assessment of the certainty of evidence | | There is moderate and limited quality of evidence in support of fluoride toothpaste and fluoride varnish for early childhood caries prevention | |
| 14. Reporting on limitations | | The main limitations with the present review were the restriction to the English language and the fact that the systematic reviews were not quality assessed.  A certain publication bias might also have occurred, as the findings from the three most recent trials in high-risk children19-21 were generally less in favor of fluoride varnish and, in fact, statistically nonsignificant | |
| 15. Reporting on recommendations | | There is moderate and limited quality of evidence in support of fluoride toothpaste and fluoride varnish for early childhood caries prevention, while the evidence for fluoride tablets/drops is insufficient. | |
| **OVERVIEW OF REVIEWS EVALUATING THE EFFECT OF PROFESSIONAL FLUORIDE VARNISH APPLICATIONS ON THE INCIDENCE OF DENTAL CARIES IN PRESCHOOLERS** | | | |
| **Mishra_2017**  Title: Role of fluoride varnish in preventing early childhood caries: A systematic review  Metanalysis: No meta-analysis conducted  Authors: Poulami Mishra, Nusrath Fareed, Hemant Battur, Sanjeev Khanagar, Manohar A Bhat, Jagan Palaniswamy  Reference and language of publication: Dent Res J (Isfahan). May-Jun 2017;14(3):169-176. doi: 10.4103/1735-3327.208766. English  Corresponding author's contact: Dr. Poulami Mishra, Department of Public Health Dentistry, KVG Dental College and Hospital, Sullia, Karnataka, India. E-mail: poulamimishra92@gmail.com  Information collected by: Flávia  Date: **08/12/2023** | | | |
| **Review Methods** | | | |
| 1. Aim | | the aim of this systematic review was to assess the role of fluoride varnish in preventing ECC. | |
| 2. Inclusion criteria | | All the published literature irrespective of study designs available over the past 36 years (1979–2015) were identified | |
| 3. Exclusion criteria | | Studies published in languages other than English were not included because of their virtual absence. Furthermore, 52 records were excluded as they were studies which included a combination of varnishes with a topical fluoride agent. | |
| 4. Bases Searched and limitations (date and language) | | PubMed/Medline (until the year 2015), Cochrane (1979–2015), EMBASE (1982–  2015) and IRIS database WHO, (until 2015). Potentially relevant reports identified from the reference lists of relevant studies, review articles, and chapters were hand-searched. Gray literature was consulted. | |
| 5. PRISMA diagram | | 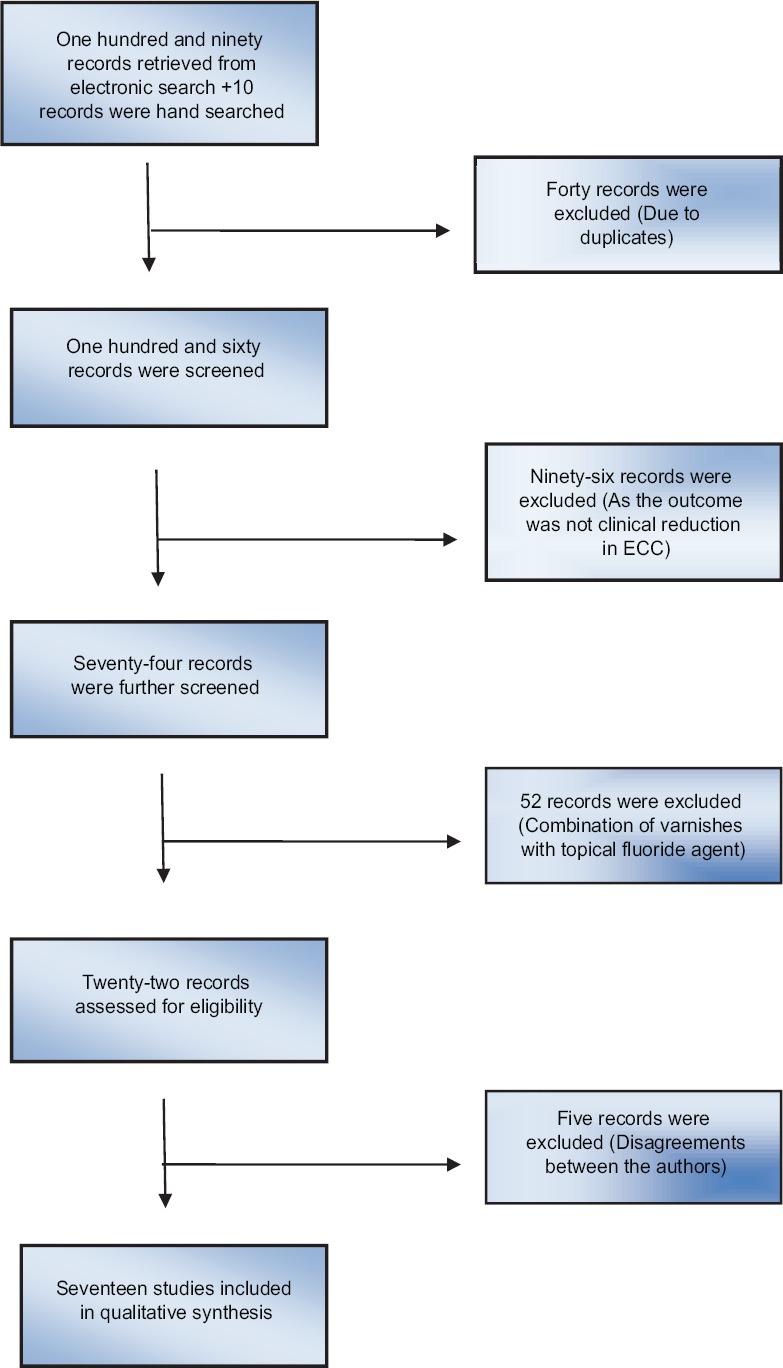 | |
| 6. Population | | children, aged 1–5 years / primary teeth | |
| 7. Intervention | | application of fluoride varnish with unavoidable fluoride exposure (fluoridated tablet, fluoridated water, and fluoridated toothpaste) | |
| 8. Comparators | | - | |
| 10. Primary and secondary outcomes | | The main outcome of our investigation was the prevention of ECC | |
| 11. Quality and bias assessment method | | JBI critical appraisal checklist for systematic reviews | |
| **Results** | | | |
| 12. Number of included studies and participants | | 17 manuscripts were included in qualitative synthesis  approximately 13,583 children aged 1–5 years were assigned to the test groups (fluoride varnish) and placebo (control groups). | |
| 13. SRs assessment of the certainty of evidence | | The studies were analyzed for levels of evidence according to JBI criteria. | |
| 14. Reporting on limitations | | No report | |
| 15. Reporting on recommendations | | this systematic review showed the evidence of studies supporting it to be of limited value.  It is recommended to conduct further studies on the effect of fluoride varnish on ECC, with improvised methodology in terms of sample size determination, randomization, blinding, the duration of the study, the use of placebos, accountability for dropouts, etc. | |

| **OVERVIEW OF REVIEWS EVALUATING THE EFFECT OF PROFESSIONAL FLUORIDE VARNISH APPLICATIONS ON THE INCIDENCE OF DENTAL CARIES IN PRESCHOOLERS** | |
| --- | --- |
| **Sousa_2019**  Title: Fluoride Varnish and Dental Caries in Preschoolers: A Systematic Review and Meta-Analysis  Metanalysis:  Authors: Fernanda Santos de Oliveira de Sousa, Ana Paula Pires dos Santos, Paulo Nadanovsky, Philippe Hujoel, Joana Cunha-Cruz, Branca Heloisa de Oliveira  Reference and language of publication: Caries Res. 2019;53(5):502-513. doi: 10.1159/000499639. English  Corresponding author's contact: Ana Paula Pires dos Santos Department of Community and Preventive Dentistry, School of Dentistry Rio de Janeiro State University, Boulevard 28 de Setembro, 157, 2o andar, Vila Isabel Rio de Janeiro, RJ 20551-030 (Brazil) E-Mail ana.paulapires @ uol.com.br or paulapires @ uerj.br  Information collected by: Flávia  Date: **15/12/23** | |
| **Review Methods** | |
| 1. Aim | to assess the effectiveness of FV in reducing the risk of developing new dentine caries  lesions and caries-related hospitalizations in preschoolers and to assess whether its effectiveness is influenced by baseline caries levels |
| 2. Inclusion criteria | Individual or cluster randomized or quasi-randomized controlled trials with a follow-up of at least 1 year. Participants were children up to 71 months of age (preschoolers). The interventions included FV – alone or associated with an oral health program – compared to placebo, usual care, or no intervention. Outcomes were caries at dentine level in the primary dentition assessed by any caries index and/or measurement of disease occurrence and hospitalizations due to caries. Short-term (allergy, itch, discomfort) and long-term (dental fluorosis) adverse effects were considered. |
| 3. Exclusion criteria | Not mentioned |
| 4. Bases Searched and limitations (date and language) | For the electronic search, the databases consulted were the Cochrane Central Register of Controlled Trials, MEDLINE via PubMed, Web of Science, EMBASE, SCOPUS, LILACS, and BBO. Sources of grey literature included meeting abstracts of the International Association for Dental Research (2001–2018) and the European Organisation for Caries Research (1998–2018), Open Grey, EThOS, the New York Academy of Medicine (GreyLit Report), and Banco de Teses CAPES. The following registers of ongoing trials were searched: Current Controlled Trials, ClinicalTrials.gov, EU Clinical Trials Register, Australia New Zealand Clinical Trials Registry, and Registro Brasileiro de Ensaios Clínicos. The search strategy was developed for MEDLINE via PubMed and adapted for the other databases and included controlled vocabulary and free terms (online suppl. Appendix 1; for all online suppl. material, see www.karger.com/doi/10.1159/000499639). References of eligible trials and systematic and narrative reviews on the subject were checked in order to detect potential studies. There were no language restraints. Hand searching was performed in nine dental journals and two medical journals (online suppl. Appendix 2) starting from the date of last update available at the Cochrane Master List of Journals Being Searched. All electronic and hand searches were last updated in July and August 2018, respectively. |
| 5. PRISMA diagram | 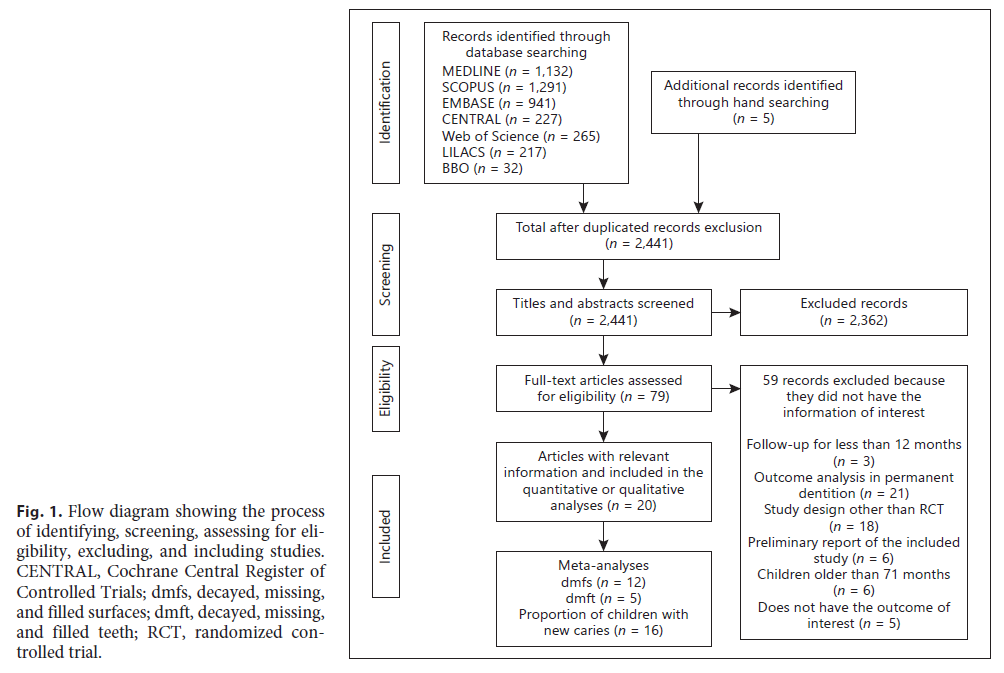 |
| 6. Population | children up to 71 months of age (preschoolers) |
| 7. Intervention | FV – alone or associated with an oral health program |
| 8. Comparators | placebo, usual care, or no intervention |
| 10. Primary and secondary outcomes | caries at dentine level in the primary dentition assessed by any caries index and/or measurement of disease occurrence and hospitalizations due to caries. Short-term (allergy, itch, discomfort) and long-term (dental fluorosis) adverse effects were considered. |
| 11. Quality and bias assessment method | We used the Cochrane risk of bias tool and the assessment included the following domains: random sequence generation, allocation concealment, blinding of participants and personnel, blinding of outcome assessment, incomplete outcome data, selective reporting, baseline balance, and diagnosis reliability. |
| **Results** | |
| 12. Number of included studies and participants | 20 studies were included: 19 in the qualitative analysis and 17 in at least one meta-analysis. The total number of children randomized was 16,877, and 13,658 were included in the analyses. |
| 13. SRs assessment of the certainty of evidence | No assessment |
| 14. Reporting on limitations | although we developed very sensitive electronic search strategies, we cannot guarantee that we were able to identify all studies that would meet our eligibility criteria.  according to our publication bias analyses, we cannot rule out the possibility of publication  bias in this review. |
| 15. Reporting on recommendations | No report |

| **OVERVIEW OF REVIEWS EVALUATING THE EFFECT OF PROFESSIONAL FLUORIDE VARNISH APPLICATIONS ON THE INCIDENCE OF DENTAL CARIES IN PRESCHOOLERS** | |
| --- | --- |
| **Yu_2021**  Title: The additional benefit of professional fluoride application for children as an adjunct to regular fluoride toothpaste: a systematic review and meta-analysis  Metanalysis:  Authors: Lintong Y, Xueqian Yu, Yueyang Li, Fengjiao Yang, Jialan Hong, Danchen Qin, Guangtai Song , Fang Hua  Reference and language of publication: Clin Oral Investig . 2021 Jun;25(6):3409-3419. doi: 10.1007/s00784-021-03909-5. Epub 2021 Mar 29. English  Corresponding author's contact: Guangtai Song [gtsong@whu.edu.cn](mailto:gtsong@whu.edu.cn)  Fang Hua [huafang@whu.edu.cn](mailto:huafang@whu.edu.cn)  Information collected by: Flávia  Date: **24/12/2023** | |
| **Review Methods** | |
| 1. Aim | The objective of this study was to assess whether the combined use of PFA and RFT has additional benefit than using RFT alone for children under 16. |
| 2. Inclusion criteria | Population (P): For consistency with existing Cochrane systematic reviews on topical fluorides, the participants of interest in this review were children aged 16 or younger at baseline (including children with deciduous, mixed or permanent dentition) [27, 28].  Intervention (I): The invention of interest was the combined use of PFA (with fluoride in any form or concentration) and RFT (≥ 1,000 ppm).  Comparison (C): The control of interest was self-applied RFT alone, with a fluoride concentration of 1,000 ppm or above.  Outcome measures (O):  The primary outcomes of this review were [29]:  (1) Increment of decayed missing/extraction indicated) and filled surfaces/teeth (D(M/E)FS or D(M/E)FT in permanent teeth and d(m/e)fs or d(m/e)ft in deciduous teeth, continuous outcome) and/or  (2) Incidence of caries (percentage of children who developed new caries, including both those of caries-free and already with caries at baseline, dichotomous outcome) and/or  (3) Changes in prevalence of caries (caries prevalence rate at follow-up minus caries prevalence rate at baseline, dichotomous outcome).  Caries was defined as being recorded at the dentine level of diagnosis. If caries data only reported caries at both dentine and enamel levels, then the data were also used in the analysis. Secondary outcomes were the progression of caries lesions through enamel or into dentine and caries arrest (that were assessed by the International Caries Detection and Assessment System (ICDAS), ICDAS II or DIAGNOdent, with continuous outcome and measured at least 6 months after application), patient-reported outcomes (e.g., ease of use/  quality of life) and fluoride-related adverse effects (e.g., dental fluorosis, allergic reactions and tooth staining).  Study design (S): RCTs with a follow-up of at least 6 months. |
| 3. Exclusion criteria |  |
| 4. Bases Searched and limitations (date and language) | We used PubMed, the Cochrane Central Register of Controlled Trials (CENTRAL), Embase and Google Scholar to search for potentially eligible articles. The search strategy was developed for PubMed and adapted for other databases (see Electronic Appendix Table 1), without any language or time restraints. In addition, the reference lists of eligible trials, as well as relevant systematic and narrative reviews, were examined. Manual searching was performed for ten relevant dental journals (see Electronic Appendix Table 2). All electronic  and manual searches were last updated in February and March 2020, respectively. |
| 5. PRISMA diagram | 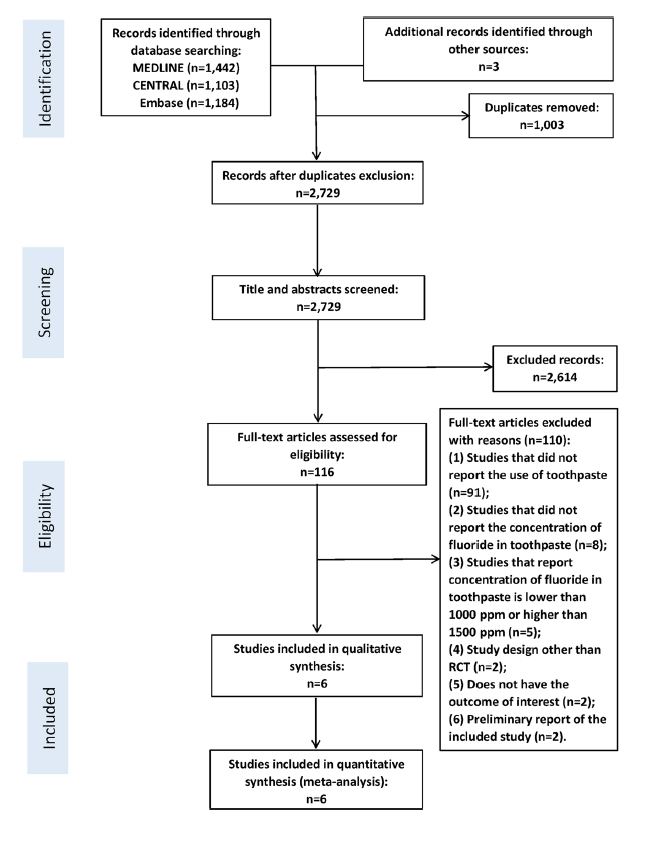 |
| 6. Population | children aged 16 or younger at baseline (including children with deciduous, mixed or permanent dentition) |
| 7. Intervention | combined use of professional fluoride application (with fluoride in any form or concentration) and regular fluoride toothpaste (≥ 1,000 ppm). |
| 8. Comparators | was self-applied regular fluoride toothpaste alone, with a fluoride concentration of 1,000 ppm or above. |
| 10. Primary and secondary outcomes | The primary outcomes of this review were [29]:  (1) Increment of decayed missing/extraction indicated) and filled surfaces/teeth (D(M/E)FS or D(M/E)FT in permanent teeth and d(m/e)fs or d(m/e)ft in deciduous teeth, continuous outcome) and/or  (2) Incidence of caries (percentage of children who developed new caries, including both those of caries-free and already with caries at baseline, dichotomous outcome) and/or  (3) Changes in prevalence of caries (caries prevalence rate at follow-up minus caries prevalence rate at baseline, dichotomous outcome).  Caries was defined as being recorded at the dentine level of diagnosis. If caries data only reported caries at both dentine and enamel levels, then the data were also used in the analysis. Secondary outcomes were the progression of caries lesions through enamel or into dentine and caries arrest (that were assessed by the International Caries Detection and Assessment System (ICDAS), ICDAS II or DIAGNOdent, with continuous outcome and measured at least 6 months after application), patient-reported outcomes (e.g., ease of use/quality of life) and fluoride-related adverse effects (e.g., dental fluorosis, allergic reactions and tooth staining). |
| 11. Quality and bias assessment method | The Cochrane risk of bias tool (V 1.0) was used to assess the ROB among included studies [33]. The tool addresses seven key domains: sequence generation, allocation concealment,  blinding of participants and personnel, blinding of assessment, incomplete outcome data, selective reporting and other biases. Two reviewers (L.Y. and X.Y.) assessed all studies independently and in duplicate,with each domain assessed as having a “high”, “low” or “unclear” risk of bias. All discrepancies were resolved by discussion with two experts (G.S. and F.H.). |
| **Results** | |
| 12. Number of included studies and participants | 5 studies and 4370 participantes up to 5 years and 1 study and 664 participants more than 6 years |
| 13. SRs assessment of the certainty of evidence | The Grades of Recommendation, Assessment, Development and Evaluation (GRADE) framework was used to assess the certainty of evidence for every primary outcome [30, 31]. Six criteria for GRADE were study design, risk of bias, precision, consistency, publication bias and other considerations. RCTs started with high certainty evidence. Thereafter, five factors (risk of bias, imprecision, inconsistency, indirectness and publication bias) might downgrade the certainty of evidence, and three factors (large effect, dose-response and all plausible confounding would reduce a demonstrated effect) may upgrade the certainty of evidence. Based on these criteria, four grades of supporting evidence (high, moderate, low or very low) were evaluated for each outcome.  Based on the GRADE assessment, the certainty of evidence for meta-analysis using the d(m/e)fs increment and incidence of caries was assessed to be moderate certainty, while the certainty of evidence for changes in prevalence of caries was found to be low |
| 14. Reporting on limitations | First, due to a limited number of relevant RCTs, there is insufficient evidence for  other types of PFA except for FV. In addition, we could not analyse the publication bias by creating funnel plots because the number of included studies was less than ten. We found little useful information about the effects of combination of RFT and FV on other clinically important outcomes, such as d(m/e)ft in the deciduous dentition and D(M/E)FS and D(M/E)FT in the permanent dentition.  We also found no useful information on adverse effects such as dental fluorosis, allergic reactions or tooth staining. Due to the lack of evidence of adverse effects, it is more difficult for clinicians and policymakers to weigh the benefit of combining FV and  RFT. |
| 15. Reporting on recommendations | No report |

| **OVERVIEW OF REVIEWS EVALUATING THE EFFECT OF PROFESSIONAL FLUORIDE VARNISH APPLICATIONS ON THE INCIDENCE OF DENTAL CARIES IN PRESCHOOLERS** | |
| --- | --- |
| **Manchanda_2021**  Title: Topical fluoride to prevent early childhood caries: Systematic review with  network meta-analysis  Metanalysis: Network Metanalisis  Authors: Sheetal Manchanda, Divesh Sardana, Pei Liu, Gillian HM Lee, Kar Yan Li , Edward CM Lo ,  Cynthia KY Yiu  Reference and language of publication: J Dent. 2022 Jan;116:103885. doi: 10.1016/j.jdent.2021.103885. Epub 2021 Nov 12. English  Corresponding author's contact: Paediatric Dentistry and Orthodontics, 2nd floor, Prince Philip Dental Hospital, 34 Hospital Road, Sai Ying Pun, Hong Kong SAR.  E-mail address: ckyyiu@hkucc.hk (C.K. Yiu).  Information collected by: Flávia  Date: **17 outubro 2023** | |
| **Review Methods** | |
| 1. Aim | synthesize the direct and indirect evidence by comparing the caries-preventive effectiveness of two or more topical forms (professionally and/or self-applied) fluorides. |
| 2. Inclusion criteria | • Population (P): Children younger than 6 years of age  • Intervention (I): Any form of topical fluoride (professionally-applied or self-applied) used for the prevention of ECC  • Control (C): Any other form/concentration of topical fluoride or placebo or no intervention  • Outcome (O): Caries increment in the primary dentition diagnosed visually and/or via tactile means was considered a primary outcome of this systematic review. Progression of caries was evaluated in the included trial as caries increment and/or caries incidence. Randomized clinical trials with a minimum of one year of follow-up were included in the review with the end-point for outcome assessment at the end of the trial. If the trial presented results in more than one manuscript, the paper with the final follow-up was considered for analysis. Furthermore, the review intended to evaluate any reported adverse effect associated with the intervention or control.  • Type of studies (S): Randomized clinical trials |
| 3. Exclusion criteria | Laboratory or in-situ studies and clinical studies with the split-mouth design were excluded. Also, studies evaluating caries through DIAGNOdent or QLF (Quantitative Light Fluorescence) were excluded due to insufficient evidence concerning the diagnostic accuracy of these methods [21,22]. The studies assessing caries arrest or reversal were  also excluded as the review focused on ECC prevention rather than treatment. Studies in which the fluoride intervention or control had additional components (like CPP-ACP or xylitol) were also excluded. The studies with full-text other than English were excluded from the review. |
| 4. Bases Searched and limitations (date and language) | Eight electronic databases, namely Medline (via Ovid), PubMed, Embase (via Ovid), Scopus, Lilacs, CINAHL, Web of Science, and Cochrane Library, were searched independently by two authors (MS and SD) up to 12th July 2020. No limitations were set regarding the year of publication or language during the search. The complete search strategy with the yields is presented in Appendix 1. The reference list of the included studies and previous published systematic reviews were searched manually to identify potential papers that could be included in this review. Additional searching was done for unpublished trials and grey literature using www.clinicaltrials.gov and <http://opengrey.eu> respectively.  Restricted to studies with full text in English |
| 5. PRISMA diagram | 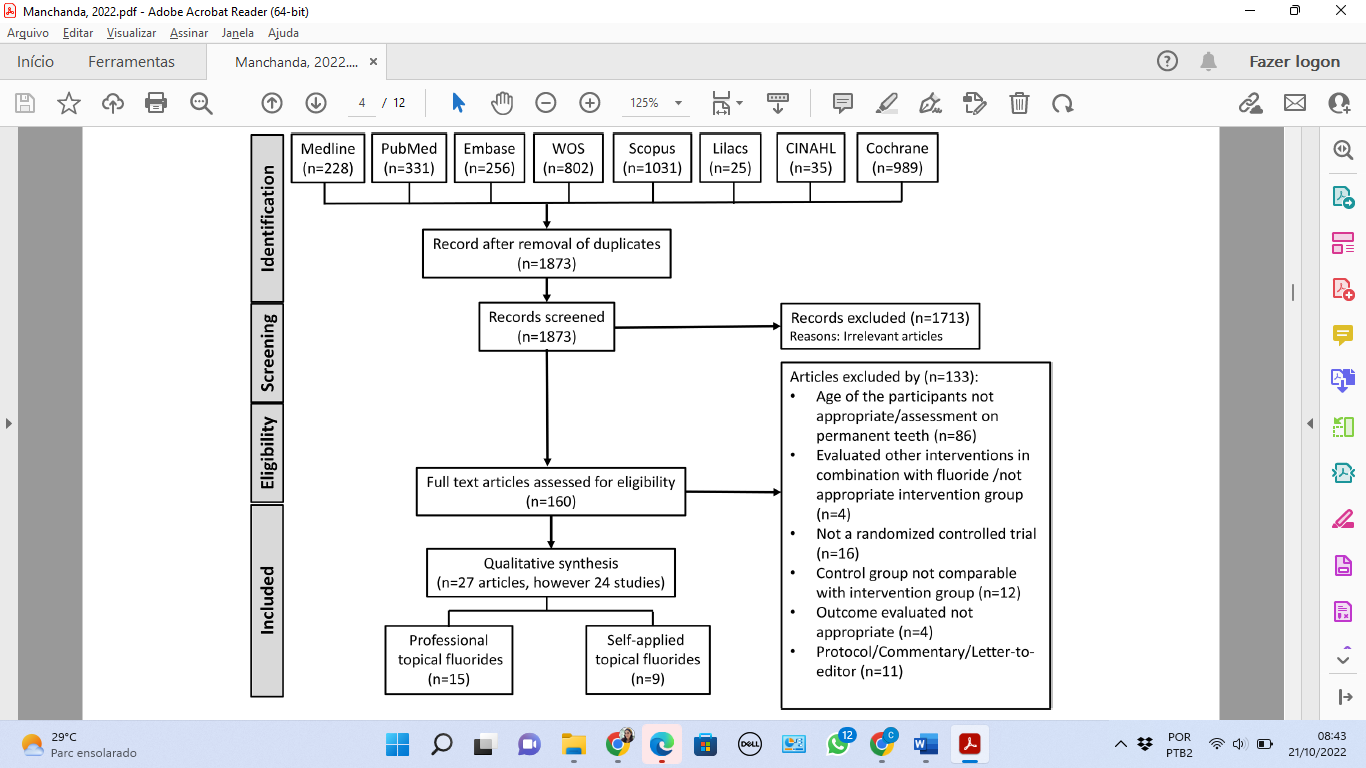 |
| 6. Population | Children younger than 6 years of age |
| 7. Intervention | Any form of topical fluoride (professionally-applied or self-applied) |
| 8. Comparators | Any other form/concentration of topical fluoride or placebo or no intervention |
| 10. Primary and secondary outcomes | Caries increment in the primary dentition diagnosed visually and/or via tactile means was considered a primary outcome of this systematic review. Progression of caries was evaluated in the included trial as caries increment and/or caries incidence. |
| 11. Quality and bias assessment method | Cochrane risk of bias tool (RoB 2.0) |
| **Results** | |
| 12. Number of included studies and participants | Twenty-four trials (published in 27 articles) included in the review were published between 1978 and 2019. The studies were conducted in 16 countries and involved 20,133 participants. |
| 13. SRs assessment of the certainty of evidence | The extension of the Grading of Recommendations Assessment, Development, and Evaluation (GRADE) approach [28] using the Confidence in Network Meta-analysis (CINeMA) framework [29] was used to generate the confidence in the evidence by two authors (MS and SD) using a web application (https://cinema.ispm.unibe.ch/). The following six domains were considered in the CINeMA framework for evaluating the level of confidence in NMA: (i) within-study bias, (ii) across-studies bias, (iii) indirectness, (iv) imprecision, (v) heterogeneity, and (vi) incoherence. The two authors assessed the level of concerns for each relative treatment effect from NMA as ‘no concerns’, ‘some concerns’, or ‘major concerns’ in each of the six domains. Subsequently, judgments across the domains were summarized into a single confidence rating (‘high’, ‘moderate’, ‘low’, or ‘very low’). The standardized mean difference (SMD) value of 0.30 was chosen for indicating the significant clinical importance that represents a small to moderate effect size [30].  Among the included studies, the confidence rating of different comparisons of various interventions generated through the CINeMA framework ranged from ‘very low’ to ‘moderate’ (Table 2). Among the 36 total comparisons in professionally-applied topical fluorides, the certainty was ‘moderate’ in 3 comparisons, which were all direct. Among these, 2 were compared with control, and one comparison was between 5% NaF varnish yearly and 5% NaF varnish 6-monthly application. The principal reason for downgrading the certainty of the evidence was due to seriousness in imprecision, within-study bias, and indirectness domains. Confidence in estimates for reporting bias was not downgraded due to the presence of symmetry and balance in the funnel plot on both |
| 14. Reporting on limitations | included studies published in English only The number of studies included in NMA is small with a limited number of arms of interventions in the network map, and thus a fixed effect model should have been used.  Another limitation occurred in the analysis of transitivity. Evaluation of the distribution of effect modifiers was impossible for assessing transitivity in this study due to the lack of enough available studies per comparison. |
| 15. Reporting on recommendations | the review recommends future good quality studies comparing two or more of these interventions with adequate follow-up to enhance the credibility of conclusions among different interventions for topical fluorides. |

| **OVERVIEW OF REVIEWS EVALUATING THE EFFECT OF PROFESSIONAL FLUORIDE VARNISH APPLICATIONS ON THE INCIDENCE OF DENTAL CARIES IN PRESCHOOLERS** | |
| --- | --- |
| **Munteanu 2022**  Title: Review of Professionally Applied Fluorides for Preventing Dental Caries in Children and Adolescents  Metanalysis: Não  Authors: Aneta Munteanu, Alina-Maria Holban, Mihaela-Rodica Pauna, Marina Imre,Alexandru-Titus Farcasiu and Catalina Farcasiu  Reference and language of publication: English  Corresponding author's contact: marina.imre@umfcd.ro (M.I.); alexandru.farcasiu@umfcd.ro (A.-T.F.)  Information collected by: Flávia  Date: **13/04/2023** | |
| **Review Methods** | |
| 1. Aim | This review aims to evaluate the effectiveness of professional topical fluoride application  on the prevention of dental caries in primary and permanent dentition. |
| 2. Inclusion criteria | Inclusion criteria:  􀀀 Participants: children and adolescents, treated in a dental care setting;  􀀀 Intervention: professionally applied fluorides (gel, varnish, foam, mouthrinse);  􀀀 Comparator: no professional treatment or other preventive treatments;  􀀀 Outcomes: clinical effectiveness (e.g., caries reduction, tooth remineralization);  􀀀 Study design: randomized controlled trials, systematic reviews, meta-analyses;  􀀀 Publication period: 1 January 2000–31 December 2021. |
| 3. Exclusion criteria | Exclusion criteria: clinical studies about home-use fluoride products, discussion papers,  in-vitro studies, case reports, non-English articles, and studies with unclear methodology |
| 4. Bases Searched and limitations (date and language) | A web search was conducted for English papers published between 2000 and 2021, using different digital resources (Pubmed, Google Scholar, Cochrane Library, and ResearchGate) |
| 5. PRISMA diagram | Not mentioned |
| 6. Population | children and adolescents, treated in a dental care setting; |
| 7. Intervention | professionally applied fluorides (gel, varnish, foam, mouthrinse) |
| 8. Comparators | no professional treatment or other preventive treatments |
| 10. Primary and secondary outcomes | caries reduction |
| 11. Quality and bias assessment method | Not mentioned |
| **Results** | |
| 12. Number of included studies and participants | 3 studies  142 + 504 + 200 = 846 participants |
| 13. SRs assessment of the certainty of evidence | No assessment |
| 14. Reporting on limitations | The limitations of this study are related to the extension of the literature search, as well  as related to characteristics of RCTs, with some of them presenting unclear randomization  methodology, a reduced number of subjects, or a short follow-up period. |
| 15. Reporting on recommendations | Reports ADA and EAPD recommendations |

| **OVERVIEW OF REVIEWS EVALUATING THE EFFECT OF PROFESSIONAL FLUORIDE VARNISH APPLICATIONS ON THE INCIDENCE OF DENTAL CARIES IN PRESCHOOLERS** | |
| --- | --- |
| **HE_2023**  Title: Clinical interventions with various agents to prevent early childhood caries: A systematic review with network meta-analysis  Metanalysis: Yes  Authors: Shuyang He, Elaine Kar Man Choong, Duangporn Duangthip,Chun Hung Chu, Edward Chin Man Lo  Reference and language of publication:  Corresponding author's contact: Correspondence Edward Chin Man Lo, Faculty of Dentistry, The University of Hong Kong, 34 Hospital Road, Sai Ying Pun, Hong Kong City, Hong Kong. Email: edward-lo@hku.hk  Information collected by: Flávia  Date: **25/04/2023** | |
| **Review Methods** | |
| 1. Aim | This review aimed to summarize and rank the effectiveness of clinical interventions using different agents for primary prevention of ECC. |
| 2. Inclusion criteria | The inclusion and exclusion criteria were as follows and organized using the Patients, Intervention, Comparison, Outcomes and Study design (PICOS) strategy:  *Participants*: Preschool children up to 72 months old, with at least one caries-free tooth (dmft = 0) and without serious systemic diseases.  *Interventions*: The interventions included prescribed products or agents applied by the children, their caregivers, dental personnel, or health-care providers to prevent ECC. There were no restrictions on the type, concentration, amount, duration, frequency of application, post application procedure, or settings.  *Comparisons*: Comparison groups in the study could be blank, placebo control, or active control. The study was eligible when both the intervention and control groups received OHE or OHC.  *Outcomes*: Two outcome measures were included in this review and meta-analysis. The first was caries increment in the primary dentition by counting the number of new decayed teeth or tooth surfaces. Change in dmft and dmfs from baseline to the end point was defined as caries increment. The second outcome measure was caries incidence (new caries in any tooth) at the child level. The assessment time for evaluating caries increment and incidence was up to 72 months of age (mainly based on the reported mean age of the participants). Only caries reported at the cavitation level (either amelodentinal or dentinal cavity) were recorded.  *Study design*: Only prospective-controlled interventional studies using parallel or split-mouth design with at least 12 months of follow-up were included. |
| 3. Exclusion criteria | The inclusion and exclusion criteria were as follows and organized using the Patients, Intervention, Comparison, Outcomes and Study design (PICOS) strategy:  *Interventions*: Interventions that only included carious teeth or selected teeth were excluded. Interventions comprised of oral health education (OHE), motivational interviewing (MI) or counselling (OHC) only were also excluded.  *Comparisons*: If only one group received the OHE or OHC intervention in the study and its effect could not be offset, the study was not included.  *Study design*:  Interventional studies using self-or historical-control were excluded. |
| 4. Bases Searched and limitations (date and language) | Three electronic databases, Medline (via PubMed), Embase (via Ovid), and Cochrane Central Register of Controlled Trials (CENTRAL), were searched at two time points (first on March 8, 2021, and later updated on June 15, 2021) without time and language limitations. The complete search strategy is presented in Appendix S1. Manual search was performed on the reference lists of the included studies and previously published reviews to  identify any potentially eligible studies. |
| 5. PRISMA diagram | 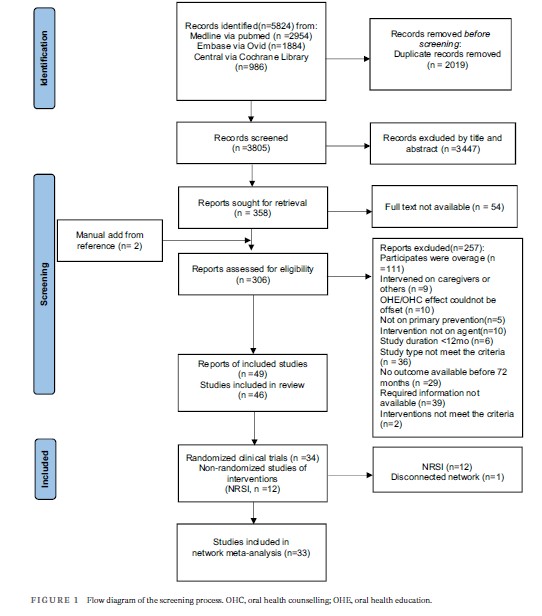 |
| 6. Population | Preschool children up to 72 months old, with at least one caries-free tooth (dmft = 0) and without serious systemic diseases. |
| 7. Intervention | prescribed products or agents applied by the children, their caregivers, dental personnel, or health-care providers to prevent ECC. |
| 8. Comparators | blank, placebo control, or active control. |
| 10. Primary and secondary outcomes | Two outcome measures were included in this review and meta-analysis. The first was caries increment in the primary dentition by counting the number of new decayed teeth or tooth surfaces. Change in dmft and dmfs from baseline to the end point was defined as caries increment. The second outcome measure was caries incidence (new caries in any tooth) at the child level. The assessment time for evaluating caries increment and incidence was up to 72 months of age (mainly based on the reported mean age of the participants). Only caries reported at the cavitation level (either amelodentinal or dentinal cavity) were recorded. |
| 11. Quality and bias assessment method | Cochrane risk of bias tool (RoB2.0) was used to evaluate the included randomized clinical trials (RCTs) in the following domains: randomization process, deviations from intended interventions, missing outcome data, measurement of outcome, and selection of reported results. The nonrandomized studies of interventions (NRSI) were assessed by the Risk of Bias in Nonrandomized Studies –of Interventions (ROBINS-I) tool, which comprised similar aspects as the RoB2.0. The highest score in any of these domains was assigned as the overall risk of bias. |
| **Results** | |
| 12. Number of included studies and participants | 49 publications (46 studies) were included |
| 13. SRs assessment of the certainty of evidence | The degree of certainty in the evidence in the NMA involving 33 RCTs ranged from “very low” to “moderate” for caries increment and from “very low” to “high” for caries  incidence, respectively (Appendix S11). Most of the comparisons were downgraded due to the issues with imprecision and bias within the included studies. |
| 14. Reporting on limitations | There are a few limitations in this systematic review.  First, the density of the network maps was unbalanced. Except for the studies on fluoride toothpaste and fluoride varnish, most of the other agents were investigated in one clinical trial only. Additionally, the SUCRA ranking presented in this paper merely rescaled the point estimates for each treatment without regard to the uncertainty or heterogeneity of the point estimates. The CINeMA assessment showed that most of the estimated results were in a very low confidence rating.  Second, in the NMA, the relationship of the included studies constituted two disconnect network maps. Although methods have been proposed to deal with disconnected network of evidence, evaluating which approach is the most appropriate often depends on the clinical context and the availability of data.77 A simpler strategy is to analyze the network maps independently. In the present NMA, the smaller network map comprised only one study64 and its result did not show any significant difference, so it was not included in the analysis. Thus, the effects of PIFG and FG with other studies were compared.  Third, in the analysis, we did not control for the dose, concentration (except for fluoride toothpaste and fluoride solution) and compound of the preventive agent, or the background fluoride exposure. These are potential effect modifiers. Not addressing these factors may break the transitivity of the NMA and confound the estimated result.  Fourth, the literature search was conducted in only three electronic databases. There is a possibility of missing relevant studies despite efforts to identify all potentially eligible studies through both electronic and manual searches.  Fifth, although pit and fissure sealants have been shown to be effective in preventing occlusal caries in primary molars78 by forming a physical barrier79 to prevent plaque and debris accumulation, it was not included in this review. This is because the outcomes of pit and fissure sealant studies were limited to primary molars only. Thus, the results were not comparable with studies investigating other agents in which outcomes relating to all teeth were reported.  Sixth, most of the studies included in our review reported results based on the per-protocol  principle. This can be a source of bias and may have resuled in an overestimation of the true effect.80  Seventh, it is worth noting that choosing the smaller SMD in caries increment would yield a more conservative result in the meta-analysis, and underestimate the caries-preventive effect of the agent. |
| 15. Reporting on recommendations | It is strongly recommended that the reports of future research should be more standardized and make all essential data available to the readers. For many of the preventive agents identified in the present systematic review, only one or two RCTs had been conducted on each of them. Additional high-quality clinical studies are required to build a stronger evidence base. Clinicians and researchers should note that, besides fluoride toothpaste and fluoride varnish, there are other choices of agents for the prevention of ECC but these agent need to be studied further. In clinical practice, there are many other factors to consider when making a recommendation for a particular child patient or a population, such as the safety, ease of use, availability, and cost of the agents. |

| **Fluoride varnish for caries prevention in preschoolers: an overview** | |
| --- | --- |
| **Rup_2023**  Title:  **CARIES INCIDENCE AFTER PROFESSIONAL FLUORIDE TREATMENT:**  **A SYSTEMATIC REVIEW**  Metanalysis: NO  Authors: Ariel Goulart Rup, Bianca Tatsch Silveira, Berenice Barbachan e Silva, Helora Freitas Moura, Jonas de Almeida Rodrigues, Juliana Jobim Jardim  Reference and idiom of publication: Goulart Rup, A., Tatsch Silveira, B., Barbachan e Silva, B., Freitas Moura, H., de Almeida Rodrigues, J., & Jardim, J. J. (2023). Incidência de cárie após tratamento profissional com fluoretos: uma revisão sistemática. *Revista Da Faculdade De Odontologia De Porto Alegre*, *64*, e127960. <https://doi.org/10.22456/2177-0018.127960>  Corresponding author's contact: Juliana Jobim Jardim - E-mail: jujobim@yahoo.com  Information collected by: Fernanda Sousa  Date: 25/07/2025 | |
|  | |
| **Review Methods** | |
| 1. Aim | the present systematic review of the literature aims to assess whether professional topical application of fluoride at high concentrations is effective for preventing new long-term injuries in both primary and permanent dentition. |
| 2. Inclusion criteria | Randomized clinical trials (RCTs) with at least 6 months of follow-up  **Types of participants**  Adolescents and children without restriction of age, gender, or ethnicity of the sampled individuals  Studies assessing both primary and permanent dentitions  Presence of enamel and/or dentin caries lesions at the beginning of the study.  **Types of interventions**  Topical fluoride application (varnish, gel, foam, or highly concentrated solution)  **Types of outcome measures**  Caries incidence, which can be measured by DMF - T/S, dmf -T/S, or ICDAS, including non-cavitated and cavitated lesions. We also assessed the caries preventive fraction (PF).  **Search methods for identification** |
| 3. Exclusion criteria | NOT MENTIONED |
| 4. Information sources (date and idiom) | Search was conducted until February 2022. (Medline via PubMed, Embase, LILACS, Cochrane Central Register of Controlled Trials, ClinicalTrials.gov) (Table 1). Free terms and Mesh terms previously recognized in each database were used in English.  There were no restrictions regarding the journal, year of publication, and language.  A manual search was carried out in the bibliographic references of selected articles after complete reading. |
| 5. PRISMA diagram | 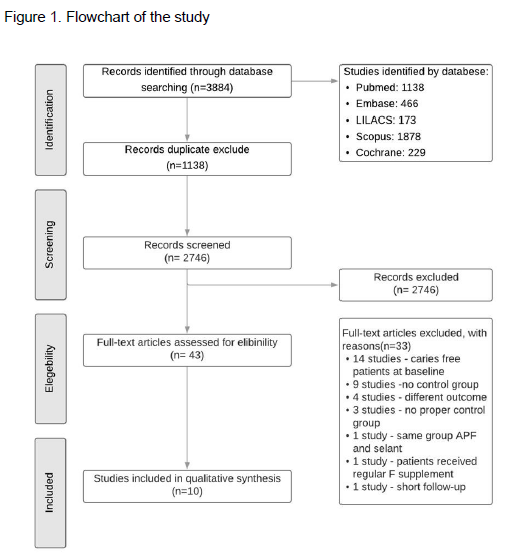 |
| 6. Population | Adolescents and children without restriction of age, gender, or ethnicity of the sampled individuals |
| 7. Intervention | Topical fluoride application (varnish, gel, foam, or highly concentrated solution) |
| 8. Comparators | No intervention or use of fluoridated toothpaste. |
| 9. Primary and secondary outcomes | Caries incidence, which can be measured by DMF - T/S, dmf -T/S, or ICDAS, including non-cavitated and cavitated lesions. We also assessed the caries preventive fraction (PF). |
| 10. Quality and risk of bias assessment method | The review presents the results of Cochrane's Risk of Bias (RoB) tool but does not provide any additional clarification. |
| **Results** | |
| 11. Number of included studies and participants | 8 studies, The studies were carried out in children and adolescents (Npeople = 5018). |
| 12. Assessment of the certainty of evidence | NOT Mentioned |
| 13. Reporting on limitations | NOT Mentioned |
| 14. Reporting on recommendations | NOT Mentioned |
