# Supplementary material for: Fluoride Varnish for Caries Prevention in Preschoolers: An Overview of Reviews
Source: Community Dent Oral Epidemiol. 2025 Nov 20;54(2):203–19. doi: 10.1111/cdoe.70032 (PMC13000968; doi:10.1111/cdoe.70032)
Supplement: Supplementary file 3 — Appendix S3: cdoe70032‐sup‐0003‐AppendixS3.docx. [file CDOE-54-203-s002.docx]

**Appendix 3:**  Distribution and Overlap of Primary Studies in Systematic Reviews: Inclusion Patterns and Citation Frequency.

| Systematic  review Primary study | n | Bader et al., 2001 | Rozier, 2001 | Petersson et al., 2004 | Azarpazhooh and Main, 2008 | Carvalho et al, 2010 | Marinho et al., 2013 | Twetman and Dhar, 2015 | Mishra et al., 2017 | Sousa et al., 2019 | Yu et al., 2021 | Manchanda et al., 2021 | Muntenau et al., 2022 | He et al., 2023 | Rup et al., 2023 |
| --- | --- | --- | --- | --- | --- | --- | --- | --- | --- | --- | --- | --- | --- | --- | --- |
| Agouropoulos et al., 2014 | 4 |  |  |  |  |  |  |  |  | x | x | x |  | x |  |
| Anderson et al., 2016 | 4 |  |  |  |  |  |  |  |  | x | x | x |  | x |  |
| Arruda et al., 2012 | 2 |  |  |  |  |  | x |  | x |  |  |  |  |  |  |
| Autio-Gold JT and Courts, 2001 | 4 |  |  |  |  | x |  |  | x |  |  |  | x |  | x |
| Borutta et al., 2006 | 1 |  |  |  |  |  |  |  |  | x |  |  |  |  |  |
| Braun et al., 2016 | 2 |  |  |  |  |  |  |  |  | x | x |  |  |  |  |
| Chu CH, 2002 | 4 |  |  |  |  | x | x |  | x | x |  |  |  |  |  |
| Clark et al., 1985 | 2 |  | x |  |  |  | x |  |  |  |  |  |  |  |  |
| Divaris K, 2013 | 1 |  |  |  |  |  |  | x |  |  |  |  |  |  |  |
| Frostell et al., 1991 | 5 |  | x |  |  | x | x |  | x | x |  |  |  |  |  |
| Grodzka et al., 1982 | 6 |  | x | x |  | x |  |  | x | x |  |  |  | x |  |
| Holm, 1979 | 8 |  | x | x |  | x | x |  | x | x |  | x |  | x |  |
| Holve, 2008 | 1 |  |  |  |  |  |  |  | x |  |  |  |  |  |  |
| Jiang, 2014 | 3 |  |  |  |  |  |  |  | x | x |  |  |  | x |  |
| Latifi-Xhemajli, 2019 | 2 |  |  |  |  |  |  |  |  |  |  | x | x |  |  |
| Lawrence et al., 2008 | 8 |  |  |  | x^a^ |  | x | x | x | x |  | x |  | x | x |
| Lo, 2001 | 1 |  |  |  |  |  |  |  | x |  |  |  |  |  |  |
| McMahon et al., 2018 | 2 |  |  |  |  |  |  |  |  | x |  |  |  | x |  |
| Memarpour et al., 2016 | 3 |  |  |  |  |  |  |  |  | x |  | x |  | x |  |
| Memarpour et al., 2015 | 4 |  |  |  |  |  |  |  | x | x |  |  |  | x | x |
| Milgrom and Tut, 2009 | 1 |  |  |  |  |  |  | x |  |  |  |  |  |  |  |
| Minah et al., 2008 | 1 |  |  |  |  |  |  | x |  |  |  |  |  |  |  |
| Muñoz-Millán, 2018 | 3 |  |  |  |  |  |  |  |  | x |  | x |  | x |  |
| Murray, 1977 | 1 |  | x |  |  |  |  |  |  |  |  |  |  |  |  |
| Oliveira et al., 2014 | 6 |  |  |  |  |  |  | x |  | x | x | x | x | x |  |
| Petersson et al., 1985 | 2 |  |  |  |  | x |  |  |  |  |  |  |  | x |  |
| Petersson, 1998 | 4 |  | x | x |  |  |  |  | x | x |  |  |  |  |  |
| Pienihakkinen and Jokela, 2002 | 1 |  |  |  | x |  |  |  |  |  |  |  |  |  |  |
| Ramos-Gomez et al., 2012 | 1 |  |  |  |  |  |  | x |  |  |  |  |  |  |  |
| Seppä, 1982 | 0 | x |  |  |  |  |  |  |  |  |  |  |  |  |  |
| Slade et al., 2011 | 4 |  |  |  |  |  |  | x | x | x |  | x |  |  |  |
| Sundell, 2013 | 1 |  |  |  |  |  |  |  |  |  |  |  |  | x |  |
| Tickle et al., 2017 | 4 |  |  |  |  |  |  |  |  | x | x | x |  | x |  |
| Twetman, 1996 | 3 |  | x |  |  | x |  |  | x |  |  |  |  |  |  |
| Weinstein, 2009 | 2 |  |  |  |  |  |  |  | x |  |  | x |  |  |  |
| Weintraub et al., 2006 | 7 |  |  |  | x | x | x |  | x | x |  | x |  | x |  |
| Yang et al., 2008 | 2 |  |  |  |  |  | x |  |  | x |  |  |  |  |  |
| Zimmer, 1999 | 1 |  |  |  |  |  |  |  | x |  |  |  |  |  |  |
| Pitchika, 2013 | 1 |  |  |  |  |  |  |  |  |  |  |  |  | x |  |
| Su, 2019 | 1 |  |  |  |  |  |  |  |  |  |  |  |  | x |  |
| Borutta, 1991 | 1 |  |  |  |  |  | x |  |  |  |  |  |  |  |  |
| Bravo, 1997 | 1 |  |  |  |  |  | x |  |  |  |  |  |  |  |  |
| Gugwad et al., 2011 | 1 |  |  |  |  |  | x |  |  |  |  |  |  |  |  |
| Hardman et al., 2007 | 1 |  |  |  |  |  | x |  |  |  |  |  |  |  |  |
| Holm, 1984 | 1 |  |  |  |  |  | x |  |  |  |  |  |  |  |  |
| Koch, 1975 | 1 |  |  |  |  |  | x |  |  |  |  |  |  |  |  |
| Liu et al., 2012 | 1 |  |  |  |  |  | x |  |  |  |  |  |  |  |  |
| Milsom et al., 2011 | 1 |  |  |  |  |  | x |  |  |  |  |  |  |  |  |
| Modeer, 1984 | 1 |  |  |  |  |  | x |  |  |  |  |  |  |  |  |
| Salazar, 2008 | 1 |  |  |  |  |  | x |  |  |  |  |  |  |  |  |
| Sköld et al., 2005 | 1 |  |  |  |  |  | x |  |  |  |  |  |  |  |  |
| Tagliaferro et al., 2011 | 1 |  |  |  |  |  | x |  |  |  |  |  |  |  |  |
| Tewari, 1984 | 1 |  |  |  |  |  | x |  |  |  |  |  |  |  |  |
| Yang et al., 2008 | 1 |  |  |  |  |  | x |  |  |  |  |  |  |  |  |

^a^ Azarpazhooh and Main, 2008 cited the unpublished date of Lawrence, previously available in parts in a conference paper.

References

Agouropoulos A, Twetman S, Pandis N, Kavvadia K, Papagiannoulis L. Caries-preventive effectiveness of fluoride varnish as adjunct to oral health promotion and supervised tooth brushing in preschool children: a doubleblind randomized controlled trial. J Dent/2014;42:1277-1283.

Anderson M, Dahllöf G, Twetman S, Jansson L, Bergenlid AC, Grindefjord M. Effectiveness of Early Preventive Intervention with Semiannual Fluoride Varnish Application in Toddlers Living in High-Risk Areas: A Stratified Cluster-Randomized Controlled Trial. Caries Res/2016;50:17-23.

Arruda AO, Senthamarai Kannan R, Inglehart MR, Rezende CT, Sohn W. Effect of 5% fluoride varnish application on caries among school children in rural Brazil: A randomized controlled trial. Community Dent Oral Epidemiol/2012;40:267-276.

Autio-Gold JT, Courts F. Assessing the effect of fluoride varnish on early enamel carious lesions in the primary dentition. J Am Dent Assoc/2001;132:53-1247.

Borutta A, Kunzel W, Rubsam F. The caries-protective efficacy of 2 fluoride varnishes in a 2-year controlled clinical trial. Deutsche Zahn Mund und Kieferheilkunde Zentralblatt/1991;79:9-543.

Borutta A, Reuscher G, Hufnagl S, Möbius S. Caries prevention with fluoride varnishes among preschool children. Gesundheitswesen/2006;68:4-731.

Braun PA, Quissell DO, Henderson WG, Bryant LL, Gregorich SE, George C, et al. A Cluster-Randomized, Community-Based, Tribally Delivered Oral Health Promotion Trial in Navajo Head Start Children. J Dent Res/2016;95:44-1237.

Bravo/1997

Chu CH, Lo EC, Lin HC. Effectiveness of silver diamine fluoride and sodium fluoride varnish in arresting dentin caries in Chinese pre-school children. J Dent Res/2002;81:70-767.

Clark DC, Stamm JW, Chin QT, Robert G. Results of the Sherbrooke-Lac Megantis fluoride varnish study after 20 months. Community Dent Oral Epidemiol/1985;13:4-61.

Divaris K, Preisser JS, Slade GD. Surface-specific efficacy of fluoride varnish in caries prevention in the primary dentition: results of a community randomized clinical trial. Caries Res/2013;47:78-87.

Frostell G, Birkhed D, Edwardsson S, Goldberg P, Petersson LG, Priwe C, et al. Effect of partial substitution of invert sugar for sucrose in combination with Duraphat treatment on caries development in preschool children: the Malmö Study. Caries Res/1991;25:10-304.

Grodzka K, Augustyniak L, Budny J, Czarnocka K, Janicha J, Mlosek K, et al. Caries increment in primary teeth after application of Duraphat fluoride varnish. Community Dent Oral Epidemiol/1982;10:9-55.

Gugwad SC, Shah P, Lodaya R, Bhat C, Tandon P, Choudhari S, et al.Caries prevention effect of intensive application of sodium fluoride varnish in molars in children between age 6 and 7 years. Journal of Contemporary Dental Practice/2011;12:13-408.

Hardman MC, Davies GM, Duxbury JT, Davies RM. A cluster randomised controlled trial to evaluate the effectiveness of fluoride varnish as a public health measure to reduce caries in children. Caries Research/2007;41:6-371.

Holm AK. Effect of fluoride varnish (Duraphat) in preschool children. Community Dent Oral Epidemiol/1979;7:5-241.

Holm GB, Holst K, Mejare I. The caries-preventive effect of a fluoride varnish in the fissures of the first permanent molar. Acta Odontologica Scandinavica/1984;42:7-193.

Holve S. An observational study of the association of fluoride varnish applied during well child visits and the prevention of early childhood caries in American Indian children. Matern Child Health J/2008;12:7-64.

Jiang EM, Lo EC, Chu CH, Wong MC. Prevention of early childhood caries (ECC) through parental toothbrushing training and fluoride varnish application: a 24-month randomized controlled trial. J Dent/2014;42:50-1543.

Koch G, Petersson LG. Caries preventive effect of a fluoride-containing varnish (Duraphat) after 1 year’s study. Community Dentistry and Oral Epidemiology/1975;3:6-262.

Latifi-Xhemajli B, Begzati A, Veronneau J,Kutllovci T, Rexhepi A, Effectiveness of fluoride varnish four times a year in preventing caries in the primary dentition: a 2 year randomized controlled trial, Community Dent. Health/2019;36:190-194.

Lawrence HP, Binguis D, Douglas J, McKeown L, Switzer B, Figueiredo R, et al. A 2-year community- randomized controlled trial of fluoride varnish to prevent early childhood caries in Aboriginal children. Community Dent Oral Epidemiol/2008;36:16-503.

Liu BY, Lo EC, Chu CH, Lin HC. Randomized trial on fluorides and sealants for fissure caries prevention. Journal of Dental Research/2012;91:8-753.

Lo EC, Chu CH, Lin HC. A community-based caries control program for pre-school children using topical fluorides: 18-month results. J Dent Res/2001;80:4-2071.

McMahon A, Wright W, Steve T, Conway D, Macpherson L. Fluoride varnish for Childsmile Nursery School attenders: randomised controlled trial. J Dent Res/2018;97:576

Memarpour M, Dadaein S, Fakhraei E, Vossoughi M. Comparison of Oral Health Education and Fluoride Varnish to Prevent Early Childhood Caries: A Randomized Clinical Trial. Caries Res/2016;50:42-433.

Memarpour M, Fakhraei E, Dadaein S, Vossoughi M. Efficacy of fluoride varnish and casein phosphopeptide-amorphous calcium phosphate for remineralization of primary teeth: a randomized clinical trial. Med Princ Pract/2015;24:7-231.

Milgrom P, Tut OK. Evaluation of Pacific Islands Early Childhood Caries Prevention Project: Republic of the Marshall Islands. J Public Health Dent/2009;69:3-201.

Milsom KM, Blinkhorn AS, Walsh T, Worthington HV, Kearney-Mitchell P, Whitehead H, et al.A clusterrandomized controlled trial: fluoride varnish in school children. Journal of Dental Research/2011;90:11-1306.

Minah G, Lin C, Coors S, Rambob I, Tinanoff N, Grossman LK. Evaluation of an early childhood caries prevention program at an urban pediatric clinic. Pediatr Dent/2008;30:499-504.

Modeer T, Twetman S, Bergstrand F. Three-year study of the effect of fluoride varnish (Duraphat) on proximal caries progression in teenagers. Scandinavian Journal of Dental Research/1984;92:7-400.

Muñoz-Millán P, Zaror C, Espinoza-Espinoza G, Vergara-Gonzalez C, Muñoz S, Atala-Acevedo C, et al. Effectiveness of fluoride varnish in preventing early childhood caries in rural areas without access to fluoridated drinking water: a randomized control trial. Community Dent Oral Epidemiol/2018;46:9-63.

Murray JJ, Winter GB, Hurst CP. Duraphat fluoride varnish: a 2-year clinical trial in 5-year-old children. Br Dent J/1977;143:7-11.

Oliveira BH, Salazar M, Carvalho DM, Falcão A, Campos K, Nadanovsky P. Biannual fluoride varnish applications and caries incidence in preschoolers: a 24-month follow-up randomized placebo-controlled clinical trial. Caries Res/2014;48:36-228.

Petersson LG, Koch G, Rasmusson CG, Stanke H. Effect on caries of different fluoride prophylactic programs in preschool children. A two year clinical study. Swed Dent J/1985;9:97-104.

Petersson LG, Twetman S, Pakhomov GN. The efficiency of semiannual silane fluoride varnish applications: a two-year clinical study in preschool children. J Public Health Dent/1998;58:57-60.

Pienihakkinen K, Jokela J. Clinical outcomes of risk-based caries prevention in preschool-aged children. Community Dent Oral Epidemiol/2002;30:50-143.

Pitchika/2013[GERMANY]

Ramos-Gomez FJ, Gansky SA, Featherstone JD, et al. Mother and youth access (MAYA) maternal chlorhexidine counseling and paediatric fluoride varnish randomized clinical trial to prevent early childhood caries. Int J Paediatr Dent/2012;22:79-169.

Salazar M. Effectiveness of Bi-Annual Fluoride Varnish Application in the Control of Dental Caries in Preschool Children: Results after 12 Months of Follow-Up. Rio de Janeiro, Brazil: Universidade do Estado do Rio de Janeiro/2008.

Seppä L, Tuutti H, Luoma, H. Three-year report on caries prevention of using fluoride varnishes for caries risk children in a community with fluoridated water. Scand J Dent Res/1982;90:89-94.

Sköld U, Petersson LG, Lith A, Birkhed D. Effect of school-based fluoride varnish programmes on approximal caries in adolescents from different caries risk areas. Caries Research/2005;39:9-273.

Slade GD, Bailie RS, Roberts-Thomson K, Leach AJ, Raye I, Endean C, et al. Effect of health promotion and fluoride varnish on dental caries among Australian Aboriginal children: results from a community-randomized controlled trial. Community Dent Oral Epidemiol/2011;39:29-43.

Su/2019 [Chinese][CHINA]

Sundell AL, Ullbro C, Koch G. Evaluation of preventive programs in high caries active preschool children. Swed Dent J. 2013;37:23-29.

Tagliaferro EP, Pardi V, Ambrosano GM, Meneghim Mde C, da Silva SR, Pereira AC. Occlusal caries prevention in high and low risk schoolchildren. A clinical trial. American Journal of Dentistry 2011;24:109–14.

Tewari A, Chawla HS, Utreja A. Caries preventive effect of three topical fluorides (1 1/2 years clinical trial in Chandigarh school children of North India). Journal of the International Association of Dentistry for Children/1984;15:71-81.

Tickle M, O’Neill C, Donaldson M, Birch S, Noble S, Killough S, et al. A Randomized Controlled Trial of Caries Prevention in Dental Practice. J Dent Res/2017;96:6-741.

Twetman S, Petersson LG, Pakhomov GN. Caries incidence in relation to salivary mutans streptococci and fluoride varnish applications in preschool children from lowand optimal-fluoride areas. Caries Res/1996;30:53-347.

Weinstein P, Spiekerman C, Milgrom P. Randomized equivalence trial of intensive and semiannual applications of fluoride varnish in the primary dentition. Caries Res/2009;43:90-484.

Weintraub JA, Ramos-Gomez F, Jue B, Shain S, Hoover CI, Featherstone JD, et al. Fluoride varnish efficacy in preventing early childhood caries. J Dent Res/2006;85:6-172.

Yang G, Lin JH, Wang JH, Jiang L. Evaluation of the clinical effect of fluoride varnish in preventing caries of primary teeth. Hua Xi Kou Qiang Yi Xue Za Zhi/2008;26:61-159.

Yang G, Lin JH, Wang JH, Jiang L. Evaluation of the clinical effect of fluoride varnish in preventing caries of primary teeth. West China Journal of Stomatology/2008;26:61-159.

Zimmer S, Robke FJ, Roulet JF. Caries prevention with fluoride varnish in a socially deprived community. Community Dent Oral Epidemiol/1999;27:8-103.
