# Supplementary material for: Fluoride Varnish for Caries Prevention in Preschoolers: An Overview of Reviews
Source: Community Dent Oral Epidemiol. 2025 Nov 20;54(2):203–19. doi: 10.1111/cdoe.70032 (PMC13000968; doi:10.1111/cdoe.70032)
Supplement: Supplementary file 4 — Appendix S4: cdoe70032‐sup‐0004‐AppendixS4.docx. [file CDOE-54-203-s001.docx]

**Appendix 4 - GRADE ASSESSMENT**

**Table 1 - GRADE assessment conducted by Overview authors**

| **Certainty assessment** | | | | | | | **№ of patients** | | **Effect** | | **Certainty** | **Importance** |
| --- | --- | --- | --- | --- | --- | --- | --- | --- | --- | --- | --- | --- |
| **№ of studies** | **Study design** | **Risk of bias** | **Inconsistency** | **Indirectness** | **Imprecision** | **Other considerations** | **Fluoride Varnish** | **Placebo, usual care, no intervention** | **Relative (95% CI)** | **Absolute (95% CI)** |  |  |
| **Sousa, 2019. proportion of children who developed new dentine caries lesions** | | | | | | | | | | | | |
| 16 | randomised trials | serious^a^ | not serious | not serious | not serious | publication bias strongly suspected | 1966/5008 (39.3%) | 1692/4365 (38.8%) | **RR 0.88** (0.81 to 0.95) | **47 fewer per 1.000** (from 74 fewer to 19 fewer) | ⨁⨁◯◯ Low |  |
| **Sousa, 2019. Number of tooth surfaces that developed dentine caries lesions.** | | | | | | | | | | | | |
| 12 | randomised trials | serious^a^ | not serious | not serious | not serious | publication bias strongly suspected |  |  | not estimable |  | ⨁⨁◯◯ Low |  |
| **Sousa, 2019. Number of primary teeth that developed dentine caries lesions.** | | | | | | | | | | | | |
| 5 | randomised trials | serious^a^ | not serious | not serious | not serious | publication bias strongly suspected |  |  | not estimable |  | ⨁⨁◯◯ Low |  |
| **Carvalho, 2012. incidence of caries, given the presence of a cavitated lesion (level of detection C2 - enamel caries, or C3 - dentine caries) in primary dentition (dmfs).** | | | | | | | | | | | | |
| 8 | randomised trials | serious^b^ | serious^c^ | not serious | not serious | publication bias strongly suspected |  |  | not pooled | see comment | ⨁◯◯◯ Very low |  |
| **Mishra, 2017. Preventive Fraction** | | | | | | | | | | | | |
| 17 | randomised trials | very serious | not serious | not serious | serious^d^ | publication bias strongly suspected |  |  | not pooled | see comment | ⨁◯◯◯ Very low |  |
| **Petersson, 2004. Preventive Fraction** | | | | | | | | | | | | |
| 3 | randomised trials | serious^e^ | not serious | not serious | serious^f^ | publication bias strongly suspected |  |  | not estimable |  | ⨁◯◯◯ Very low |  |
| **Azaepazhooh, 2008. Effectiveness of fluoride varnish** | | | | | | | | | | | | |
| 3 | randomised trials | serious^b^ | not serious^g^ | not serious | serious | publication bias strongly suspected |  |  | not estimable |  | ⨁◯◯◯ Very low |  |
| **Rozier, 2001. Effectiveness of fluoride varnish** | | | | | | | | | | | | |
| 7 | randomised trials | serious^b^ | serious | not serious | serious | publication bias strongly suspected |  |  | not estimable |  | ⨁◯◯◯ Very low |  |
| **Bader, 2001.** **Incidence of new coronal carious lesions in primary teeth** | | | | | | | | | | | | |
| 1 | randomised trials | very serious^h^ | not serious | not serious | not serious | none |  |  | not estimable |  | ⨁⨁◯◯ Low |  |
| **Munteanu, 2022 Caries reduction** | | | | | | | | | | | | |
| 5 | randomised trials | serious^i^ | serious^g^ | not serious | not serious | publication bias strongly suspected |  |  | not estimable |  | ⨁◯◯◯ Very low |  |
| **Rup, 2023 Caries reduction** | | |  |  |  |  |  |  |  |  |  |  |
| 3 | randomised trials | serious | serious | not serious | very serious | publication bias strongly suspected |  |  |  |  | ⨁◯◯◯ Very low |  |

**CI:** confidence interval; **RR:** risk ratio

#### Explanations

a. Most studies had unclear or high risk for random sequence generation, Allocation concealment, blinding of participants and personnel, incomplete outcome data and selective reporting

b. Lack of Blinding, Incomplete accounting of patients and outcome events and selective reporting

c. Heterogeneity

d. Wide range of preventive fractions identified

e. Avaliados por um critério diferente. 2 na pior qualidade e um intermediário. Não tem sensibilidade, diferentes pesos. Além disso não é possível identificar o que aumentou o risco de vies dos estudos, falta mt informação

f. Wide range of increment values and lack of confidence intervals

g. Reviewers were unable to adjust data for comparison

h. Studies with several design issues

i. Allocation concealment, blinding of participants and personnel, selective reporting

**Table 2 - GRADE assessment extracted from the included Systematic reviews**

| **Systematic Review** | **Outcomes** | **Certainty of the evidence** | | |  |
| --- | --- | --- | --- | --- | --- |
|  |  | **GRADE** | **CINeMA** | |  |
| Yu, 2021 | Incidence of caries | ⊕⊕⊕⊝  Moderate^a^ |  |  | a Downgraded 1 level for imprecision (95% confidence interval around the pooled estimate of effect included both: no effect and appreciable benefit.) b Downgraded 1 level due to single study at unclear risk of bias (Braun 2016). c Downgraded 1 level for imprecision (there were insufficient number of participants in single study leads to a wide confidence interval (Braun 2016)) |
|  | Changes in prevalence of caries | ⊕⊕⊝⊝  Low ^ab^ |  |  |  |
|  | d(m/e)fs increment | ⊕⊕⊕⊝  Moderate ^c^ |  |  |  |
| Twetman, 2015 | reduce the incidence of early childhood caries | ⊕⊕⊝⊝  Low |  |  |  |
| Marinho, 2013 | Deciduous tooth surfaces d((e)/m)fs increment PF - nearest to 3 years (10 trials) | ⊕⊕⊕⊝  Moderate |  |  | The quality of the evidence was downgraded due to considerable heterogeneity; 5 trials were at high and 5 trials at unclear risk of bias. However this body of evidence showed a consistent, large clinically important effect and we have upgraded the quality of evidence to moderate |
| He, 2023 | Caries increment in the primary dentition by counting the number of new decayed teeth or tooth surfaces  Caries incidence (new caries in any tooth) at the child level. |  | ⊕⊝⊝⊝  Very low | ⊕⊕⊕⊝  Moderate ^c^ | Most of the comparisons were downgraded due to the issues with imprecision and bias within the included studies. |
| Manchanda, 2021 | Caries increment in the primary dentition diagnosed visually and/or via tactile |  | ⊕⊝⊝⊝  Very low | ⊕⊕⊝⊝  Low |  |

d(m/e)fs = Decayed, Missing/ should be extracted, Filled Surfaces ; PF= Prevented Fraction
