# Supplementary material for: Fluoride Varnish for Caries Prevention in Preschoolers: An Overview of Reviews
Source: Community Dent Oral Epidemiol. 2025 Nov 20;54(2):203–19. doi: 10.1111/cdoe.70032 (PMC13000968; doi:10.1111/cdoe.70032)
Supplement: Supplementary file 5 — Appendix S5: cdoe70032‐sup‐0005‐AppendixS5.docx. [file CDOE-54-203-s003.docx]

Appendix 5. Limitations, recommendations, and conclusions reported in the included systematic reviews.

| **Author, year** | **Limitations** | **Recommendations** |
| --- | --- | --- |
| Bader et al., 2001 | The scarcity of studies on primary teeth, the identification of at-risk subjects, and various issues related to the design of the studies are limitations identified. The studies varied in the product and concentration used, as well as application frequencies. | **Recommendations for research**  Increase the number of studies that examine prevention among high-risk individuals and non-surgical management of non-cavitated lesions. Evaluate a wider variety of subject ages. Targeting aspects of the efficacy questions not yet addressed. Improve studies methodologies and the reporting of methods and outcomes. |
| Rozier, 2001 | This review does not include use of combinations of professionally applied preventive methods. | **Recommendations for practice** Insufficient evidence of effectiveness to recommend for or against its use in the preschool-aged child. |
| Petersson et al., 2004 | No report on limitations | **No report on recommendations** |
| Azarpazhooh and Main, 2008 | No report on limitations | **Recommendations for practice** Fluoride varnish should be applied twice a year for predominantly high-risk populations, unless an individual's past and current caries history indicates no risk of caries. |
| Carvalho et al, 2010 | The design problems in the reviewed clinical studies should be noted. Some issues, including data asymmetry and the absence of standard deviation information, prevented the authors from conducting a meta-analysis. Data couldn't be transformed for symmetry improvement, as raw data was unavailable. Meta-regression wasn't used to explore study heterogeneity sources for the same reason. | **Recommendations for research** It is recommended to conduct well-designed randomized clinical trials to assess the anticaries benefits of fluoride varnish. |
| Marinho et al., 2013         Marinho et al., 2013 (Continuing) | No clear relationship between prevented fraction and precision could be observed in the funnel plot of the 13 trials (it appeared asymmetric), but as for meta-regression methods, power is limited when the number of trials is small. We cannot eliminate the possibility that bias may have influenced the results of this review. | **Recommendations for practice**  The application of fluoride varnishes two to four times a year is associated with a substantial reduction in caries increment. The review does not provide any information on the likelihood of side effects with this treatment and inconclusive information on acceptability.  **Recommendations for research**  There is still a paucity of evidence from high quality randomised trials assessing the effectiveness of fluoride varnishes for the prevention of caries in children. It is also important that future trials should include the assessment of other relevant outcomes such as potential side effects (e.g. oral allergic reactions) and those related to acceptability of treatment. The reporting of caries at both the cavitated and non-cavitated level would improve interpretation. Also future trials should consider evaluating the effects of complex interventions incorporating fluoride varnish with other caries preventive strategies, conducted in either the setting of a dental practice or a community site such as a school. |
| Twetman and Dhar, 2015 | The main limitations of the review include the English language restriction and potential publication bias. The three most recent trials on high-risk children were less favorable of fluoride varnish with no statistically significant results. | **Recommendations for practice**  Evidence of moderate and limited quality supports the use of fluoride varnish for preventing early childhood caries.  **Recommendations for research**  The results emphasize the need for high-quality clinical research and highlight knowledge gaps for future studies. |
| Mishra et al 2017 | No report on limitations | **Recommendations for research**  Further studies on fluoride varnish and ECC are recommended, with improved methodology, including sample size, randomization, blinding, study duration, placebo use, and dropout accountability. |
| Sousa et al., 2019 | Despite employing very sensitive electronic search strategies, the authors cannot guarantee that all eligible studies were identified. The potential for publication bias in this review cannot be ruled out according to the publication bias analyses. | **Recommendations for research** Cost-effectiveness analyses are necessary to decide on fluoride varnish (FV) use in dental services. |
| Yu et al.,2021 | The authors could not analyse the publication bias due to the limited number of studies | **No report on recommendations** |
| Manchanda et al.,2021 | Non-English studies were not included in the review. The network map in the NMA included only a small number of studies and a limited range of intervention arms, which made it advisable to use a fixed-effect model. Due to the insufficient number of studies available per comparison, it was not possible to evaluate the distribution of effect modifiers for assessing transitivity in this study. | **Recommendations for practice**  The 0.9% DFS varnish applied every 3 months is most effective for preventing early childhood caries.  **Recommendations for research**  The review calls for future high-quality studies comparing professionally applied fluorides with longer follow-up to strengthen the reliability of their conclusions. |
| Muntenau et al., 2022 | The study limitations include the extent of the literature search and some characteristics of the RCTs such as unclear randomization methods, a limited number of participants, or a short follow-up period. | **Recommendations for practice**  The review highlights recommendations from ADA and EAPD. ADA recommends two applications per year for children with moderate caries risk and two to four applications for children at high risk of caries. EAPD moderately recommends two to four applications of fluoride varnishes for caries prevention, especially in children of increased risk of caries development and children with special oral health care needs. |
| He et al., 2023 | The SUCRA ranking did not consider uncertainty or heterogeneity of estimates and only adjusted point estimates. The CINeMA assessment found most results had a very low confidence rating. Authors did not control for the following potential effect modifiers in the analysis, such as the dose, concentration, and composition of the preventive agent, as well as background fluoride exposure. This may break the transitivity of the NMA and confound the estimated result. The literature search was limited to just three electronic databases so relevant studies might be missing. Most of the studies in the review reported results based on the per-protocol principle. This could potentially overestimate the true effect. Choosing the smaller Standardized mean differences in caries increment would yield a more conservative result in the meta-analysis, and underestimate the caries-preventive effect of the agent. | **Recommendations for research**  Reports of future research should adhere to standardized reporting practices and ensure that all essential data are readily accessible to readers. High-quality clinical studies are needed to enhance the strength of evidence. |
| Rup et al., 2023 | No report on limitations | **No report on recommendations** |
